# Advances in Mass Spectrometry of Gangliosides Expressed in Brain Cancers

**DOI:** 10.3390/ijms25021335

**Published:** 2024-01-22

**Authors:** Maria Roxana Biricioiu, Mirela Sarbu, Raluca Ica, Željka Vukelić, Svjetlana Kalanj-Bognar, Alina D. Zamfir

**Affiliations:** 1 National Institute for Research and Development in Electrochemistry and Condensed Matter, 300224 Timisoara, Romania; maria.biricioiu99@e-uvt.ro (M.R.B.); mirela.sarbu86@yahoo.co.uk (M.S.); raluca.ica@gmail.com (R.I.); 2 Faculty of Physics, West University of Timisoara, 300223 Timisoara, Romania; 3 Department of Chemistry and Biochemistry, School of Medicine, University of Zagreb, 10000 Zagreb, Croatia; zeljka.vukelic@mef.hr; 4 Croatian Institute for Brain Research, School of Medicine, University of Zagreb, 10000 Zagreb, Croatia; svjetlana.kalanj.bognar@mef.hr; 5 Department of Technical and Natural Sciences, “Aurel Vlaicu” University of Arad, 310330 Arad, Romania

**Keywords:** brain cancers, gangliosides, mass spectrometry, biomarker discovery, screening, structural analysis

## Abstract

Gangliosides are highly abundant in the human brain where they are involved in major biological events. In brain cancers, alterations of ganglioside pattern occur, some of which being correlated with neoplastic transformation, while others with tumor proliferation. Of all techniques, mass spectrometry (MS) has proven to be one of the most effective in gangliosidomics, due to its ability to characterize heterogeneous mixtures and discover species with biomarker value. This review highlights the most significant achievements of MS in the analysis of gangliosides in human brain cancers. The first part presents the latest state of MS development in the discovery of ganglioside markers in primary brain tumors, with a particular emphasis on the ion mobility separation (IMS) MS and its contribution to the elucidation of the gangliosidome associated with aggressive tumors. The second part is focused on MS of gangliosides in brain metastases, highlighting the ability of matrix-assisted laser desorption/ionization (MALDI)-MS, microfluidics-MS and tandem MS to decipher and structurally characterize species involved in the metastatic process. In the end, several conclusions and perspectives are presented, among which the need for development of reliable software and a user-friendly structural database as a search platform in brain tumor diagnostics.

## 1. Introduction

Gangliosides represent a class of glycosphingolipids that are mainly located in the outer layer of the plasma membrane and possess remarkable functions in the mammalian central nervous system (CNS) [1,2,3]. A ganglioside molecule encompasses a hydrophilic *O*-glycan sequence and a hydrophobic lipid part, i.e., the ceramide (Cer) moiety, which contains a sphingoid base and a fatty acid chain.

**Scheme 1:**
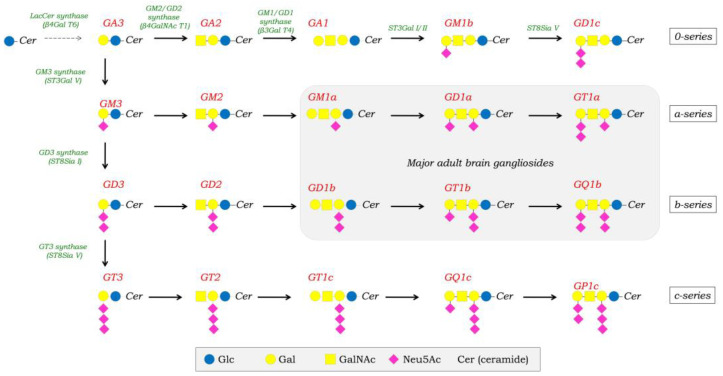
Biosynthesis pathway of gangliosides.

Similarly to other glycosphingolipids, the synthesis of gangliosides is initiated in the endoplasmic reticulum. Further on, the molecule is elongated by stepwise addition to the ceramide of monosaccharide building blocks (Scheme 1) in a reaction that is catalyzed by specific glycosyltransferases [4]. The ceramide anchors the entire molecule into the cell membrane, while the glycan chain remains free to mediate the interactions of ganglioside with soluble extracellular molecules and with hydrophilic segments of other membrane components [5].

The element distinguishing gangliosides from other types of glycosphingolipids and conferring them specific properties is sialic acid. The sugar chain of all gangliosides is decorated with one or more *N*-acetylneuraminic (Neu5Ac) or *N*-glycolylneuraminic (NeuGc) acid residue(s), attached to the oligosaccharide backbone by an aketosidic linkage. Due to this particular structure, gangliosides contribute to the cellular lipidome as well as the glycome and sialome [6]. Both the ceramide and the sialoglycan parts of the molecule present variability of the length, composition and structure, having a specificity that depends on the cell and tissue type. Moreover, the structural modifications that might occur on gangliosides, including most commonly fucosylation (Fuc), *O*-acetylation (*O*-Ac), or less commonly, de-*N*-acetylation, sulfation, or attachment of a glucuronic acid (GlcA) or N-Acetylgalactosamine (GalNAc) residue, drastically change the biophysical properties, functions and activity of the ganglioside molecule.

Although, gangliosides have been found to be expressed in all vertebrate cells, studies conducted in last two decades employing advanced biochemical and biophysical techniques have reported that the concentration of these molecules in the CNS, where they are mainly associated with synaptic membranes, is several times higher than in extraneural tissue [3]. This aspect, which suggested that gangliosides might play a particular role at the CNS level, triggered the investment of considerable efforts into the investigation of their expression and role in human brain. The follow-up results confirmed that in a normal human brain, the ganglioside pattern presents a marked topographical, phylogenetic and age-related specificity. Some gangliosides were revealed as structures crucially involved in the fundamental processes related to human brain development, maturation, and aging as well as to the upholding of the specific functions of each brain region, while others were proven as key factors in neurodegenerative disease [7] or owners of anti-tumorigenic features able to contribute to the defense mechanisms against tumors [8]. Hence, a number of species are regarded as promising therapeutic targets for inclusion in future immunotherapy schemes. On the other hand, some gangliosides are involved in a plethora of biological events induced by their functional interactions with different molecules. Gangliosides were shown to interact with signal transducers, mediate carbohydrate-dependent cell adhesion, induce cell activation, motility and growth and participate in cell–cell and cell–matrix interactions [9,10]. The growing interest in the study of ganglioside interactions with peptides and proteins was stimulated by findings indicating that the formed complexes might play a role in: (i) the molecular mechanisms of Alzheimer disease (AD), with an emphasis on the functions of amyloid β-protein-ganglioside complexes [11]; (ii) the action of bacterial toxins for which several gangliosides were found to be specific receptors [12]; (iii) the progression of malignant brain tumors [13] and (iv) the discovery of novel species in complex mixtures based on their high binding affinity [14].

Systematic investigations of gangliosides in neurological and neurodegenerative diseases, as well as primary and secondary brain tumors, disclosed significant differences in their expression as compared to the healthy brain [15]. Additionally, many structures were found to be valuable biochemical markers not only for early detection and prognosis of the disease but also for exploitation as possible therapeutic targets or agents. Mutations in ganglioside biosynthetic enzymes were found to result in severe neurodegenerative disorders, often characterized by very early or childhood onset [16]. Moreover, significant changes in the ganglioside composition and structure or in the relative abundance of specific components were reported not only in healthy developing and aging brains, but also in common neurological conditions [17,18,19], including Huntington’s disease (HD), AD, Parkinson’s disease (PD), amyotrophic lateral sclerosis (ALS), stroke, multiple sclerosis and epilepsy.

In the research of brain-associated gangliosides, traditionally thin-layer chromatography (TLC) [20,21] and immunohistochemical techniques were used for localization of gangliosides in peripheral [22] and central nervous system [23,24], and cerebellum [25], and in particular GD3 and GD2 gangliosides in cells of human intracranial tumors [26]. Since gangliosides are readily embedded on hydrophobic surfaces, their comparative profiles in various normal brain regions of different ages or in brain conditions as well as their interactions were extensively studied by specific and compatible biochemical and biophysical methods, such as high performance thin layer chromatography (HPTLC), enzyme-linked immunosorbent assay (ELISA), or the modern single-fluorescent-molecule imaging [5,27] and atomistic molecular dynamics simulations [28]. Flow cytometry [29] or nanocube-based lipid bilayer arrays [30] were also implemented for the determination of ganglioside-cholera toxin complexes, which, hitherto, is the most studied interaction in gangliosidomics. Even though these methods contributed to the advancement of the field, they present a number of drawbacks: (i) the inadequate sensitivity, which only allows collection of information on the major species in the heterogeneous native ganglioside extracts; (ii) the impossibility to structurally characterize in details the individual species and (iii) the limited data on the minor components, which frequently represent molecular markers.

Due to the sensitivity, reproducibility, data accuracy, wealth of structural information and the compatibility with aqueous solutions, in mid-1980s MS started to become a method of choice in human brain ganglioside research. The first successful approach employed fast-atom bombardment (FAB) MS with encouraging results [31], despite the challenges related to the low ionization efficiency and the high structural complexity, which make gangliosides much less amenable to MS than other biomolecules. The development, a few years later, of nanoelectrospray (nanoESI) and matrix-assisted laser desorption/ionization (MALDI) methods represented the factual breakthrough of mass spectrometry in human brain ganglioside analysis [32,33,34]. With the succeeding technical innovations, such as microfluidics-MS [34,35,36,37,38], the high resolution analyzers, such as Fourier transform ion cyclotron resonance (FTICR) [39,40], Orbitrap [41,42], ion mobility separation (IMS) MS [43,44] as well as desorption electrospray (DESI) and MALDI MS imaging (MSI) [45,46,47] and the efficient ion fragmentation methods [48], the achievements of mass spectrometry in profiling, detailed structural analysis and discovery of normal and pathological brain-associated gangliosides increased spectacularly. In this context, the present review discusses the trends in the important field of brain cancer ganglioside analysis using modern MS approaches, highlighting the invaluable contributions of this method in establishing well-defined sets of biomarkers and elucidating some of the ganglioside-dependent mechanisms of tumor proliferation.

## 2. Primary Brain Tumors

### 2.1. Benign Tumors

Vascular anomalies encompass a group of conditions marked by irregularities in the development or expansion of blood and/or lymphatic vessels. These diseases exhibit a diverse range of complications and severity levels [49]. Their diagnostics can be intricate due to the diversity of the clinical presentations and the overlap in symptoms among such disorders. Individuals affected by these anomalies often seek care from different medical and surgical specialties, therefore, ensuring an accurate diagnostic and employing shared terminology are crucial to enabling a comprehensive evaluation and effective management of these conditions.

Vascular malformations can have a profound impact on patients, leading to a variety of clinical issues that significantly affect their quality of life. The symptoms encompass a wide spectrum, including disfigurement, acute and chronic pain, coagulopathy, bleeding, thrombosis, as well as dysfunction of various organs and the musculoskeletal system. In the most severe cases, these disorders exhibit a progressive nature and often result in lifelong complications [50]. The challenges they pose can be far-reaching, underscoring the importance of timely diagnostics and effective management to enhance the well-being of those affected [51].

Hemangiomas are frequently encountered as benign vascular tumors, typically making their appearance during childhood. Although a significant proportion of hemangiomas are small and relatively benign, amenable to conservative management, some may raise concerns due to their association with underlying syndromes or potential involvement of vital organs [52].

Hemangiomas can be observed in various locations, including the skin and internal organs like the brain, liver, kidney, eyes, lungs, bones, spleen, or pancreas [53,54,55]. Symptomatic hemangiomas can give rise to various complications, including ulceration, bleeding, impairments in vision, and limitations in functionality. The management of a symptomatic hemangioma often involves a multifaceted approach, with the choice of treatment contingent on factors like the tumor size, location, and proximity to critical anatomical structures. Ensuring a tailored and comprehensive treatment strategy is essential to address the specific needs and challenges presented by each individual case.

Cavernous hemangioma, the predominant form of hemangioma, is frequently found in the brain and is characterized by blood-filled cavities encased by extremely thin vascular walls. The majority of cavernous hemangiomas develop without a clear pattern and typically involve blood circulation at low pressure, contributing to a positive prognosis for affected individuals [56,57]. Among the most prominent complications associated with this tumor are hemorrhages, strokes, epilepsy, and focal neurological deficits. Such malformations are closely linked to the presence of loss-of-function mutations in one of the three genes: *KRIT1* (Krev interaction trapped 1, also referred to as *CCM1*), *CCM2*, or *PDCD10* (programmed cell death 10, also known as *CCM3*) [58,59]. This premise gains substantial support from the fact that all three proteins are typically co-located within the same cellular complex. The close association between the *CCM* genes and their coexistence in a complex underscores their interdependent roles in the development and manifestation of *CCMs*, forming a key piece of the puzzle in understanding this complex medical condition.

Over the past years, due to the advancements in imaging technology, modern medical sciences have developed various techniques and protocols to diagnose and investigate hemangiomas, among which are: (i) Computer Tomography (CT scan), a non-invasive method primarily suitable for detecting large tumors; (ii) Nuclear Magnetic Resonance (NMR) and Magnetic Resonance Imaging (MRI), able to reveal the extent of the lesions and detect potential blood vessel ruptures (hemorrhages) and (iii) ultrasonography (ultrasound), which serves as a valuable tool for distinguishing hemangiomas from other tissue alterations like cysts or lymph nodes. In addition to the imaging methods for the diagnosis of cavernous hemangiomas, new techniques, based on MS for the identification of molecular markers, were developed. As molecules differentially and specifically expressed in diseased vs. normal brain tissue, gangliosides represent those diagnostic biomarkers not only in brain cancers, but also in neurodegenerative diseases [60,61].

In this context, our group developed and introduced an advanced microfluidics system based on nanoESI chip MS methodology [62] for ganglioside biomarker discovery in human brain cavernous hemangioma. The research was carried out on a NanoMate robot (Advion Biosciences) online coupled to a high capacity ion trap MS (HCT MS) instrument to yield an advanced and highly sensitive bioanalytical platform able to explore the gangliosides expressed in hemangioma tissue. The nanoESI chip HCT MS system was configured and optimized to operate in negative ion mode [62,63,64,65]. Specifically, the analysis was focused on samples obtained from a 42-year-old male patient diagnosed using CT and MRI with a cerebral hemangioma located in the right hemisphere of the frontal cortex (HFC). The brain tumor specimen was collected during a surgical procedure, and the diagnosis of cavernous hemangioma was confirmed by histopathological examination using hematoxylin and eosin staining. As a control, a specimen from a normal frontal cortical brain tissue (NFC) sampled from a male subject of similar age who died in a traffic accident was used. Altogether, by chip-based nanoESI analysis, 29 different ganglioside species were for the first time found directly linked to cavernous hemangioma. Moreover, nanoESI chip MS screening indicated that the gangliosidome of the frontal cortex hemangioma is characterized by the prevalence of shorter, monosialylated gangliosides, among which *O*-Ac-GM4 and *O*-Ac-GD2, in contrast to the normal tissue, which showed a larger variety of gangliosides, containing from mono- to polysialylated structures. Interestingly, the presence of *O*-Ac-GD2 in HFC appeared to correlate with a lower degree of malignancy.

The distinct ganglioside pattern observed in hemangioma, as compared to healthy control tissue, may be a consequence of variations in the overall biosynthetic rate and could originate from changes in the expression of specific glycosyltransferases. The detailed structural analysis of individual species carried out by tandem MS (MS/MS) using collision-induced dissociation (CID) revealed the unique expression in HFC of the GT1c (d18:0/20:0) isomer. This interesting structural feature was further substantiated by the CID MS/MS analysis of ions related to GD1 (d18:0/20:0), which generated findings that emphasized the complex and specific ganglioside profile associated with hemangioma and provided valuable insights into the primary molecular dynamics of this condition.

More recently, the capabilities of MS for performing reliable glycolipidomic assays have advanced significantly due to the advent of versatile high-resolution (HR) MS instruments, including orbital traps. These instruments not only provide exceptional resolving power, but also allow sequencing of complex ionic species by efficient fragmentation techniques in multistage MS (MS^n^) experiments. In the particular field of brain gangliosides, the use of HR MS and MS/MS on Orbitrap instruments coupled with nanoESI has facilitated the direct identification of glycan panels and isoforms that serve as biomarkers. This advanced approach offers ultra-high resolution, precise mass accuracy, and impressive levels of sensitivity, capable of detecting quantities of analytes at concentrations down to picomoles and subpicomoles [15,66].

Therefore, in another investigation conducted by our group [67], the gangliosides associated with human brain hemangioma were re-evaluated by the HR MS approach using an Orbitrap mass spectrometer equipped with nanoESI, fine-tuned for the detection of negative ions. The experiments were carried out using an LTQ Orbitrap Velos Pro TM instrument (Thermo Fisher Scientific, Waltham, MA, USA) equipped with an offline nanoESI source. The ganglioside mixture extracted and purified from hemangioma tissue was directly infused into the instrument through the offline nanoESI source, connected to the mass spectrometer using a Nanospray Flex Ion Source provided by Thermo Scientific.

As visible in Table 1, the MS analysis of cavernous hemangioma gangliosides revealed a total of 62 distinct ions, which were accurately identified following exact mass calculations, each corresponding to specific ganglioside species. These ions were assigned to 52 structures, showcasing the remarkable diversity of these glycosphingolipids in hemangioma tumor. In comparison to the previous study on the human brain hemangioma ganglioside pattern, performed by fully automated nanoESI chip HCT MS [59], where 29 species could be detected, the high resolution of the Orbitrap instrument allowed the discrimination, identification, and thus correlation with human hemangioma, of almost double the number of ganglioside species.

Within this diverse set of gangliosides, several patterns of sialylation emerged. The gangliosides were categorized into various classes based on their sialic acid content, revealing a range of structures and functions. Notably, one ganglioside was found to be asialylated, belonging to the GA1 class. Asialo gangliosides play crucial roles in various cellular processes, and their identification in this tumor provides valuable biological relevance. Monosialylated gangliosides were found to be dominant, with a total of 14 species identified. Among these, eight belonged to the GM1 class, which is known for its implication in neuronal function and synaptic signaling [7,68]. Additionally, one ganglioside was identified as GM2, four as GM3, and one as GM4, each contributing to the complexity of hemangioma gangliosidome.

The most prominent sialylation pattern observed in this analysis was disialylation, with 23 gangliosides falling into this category. Within the disialylated group, eight were classified as GD1, four as GD2, and a remarkable number of eleven as GD3. The presence of GD3 is particularly noteworthy, as it is considered a precursor for various complex gangliosides implicated in a range of cellular processes and brain developmental stages.

The detailed analysis also revealed 13 trisialylated species, with ten belonging to the GT1 class and three to the GT3. The trisialylated structures are known to play vital roles in cellular recognition and signaling processes, contributing to the language of cell-to-cell communication [69]. Intriguingly, only one tetrasialylated ganglioside was identified, belonging to the GQ1 class [67]. This ganglioside, with its high degree of sialylation, is relatively rare, yet holds specific biological significance in certain contexts. Notably, no structures with a higher degree of sialylation were discovered in hemangioma, highlighting once more the limits of sialylation within this particular tumor.

**Figure 1:**
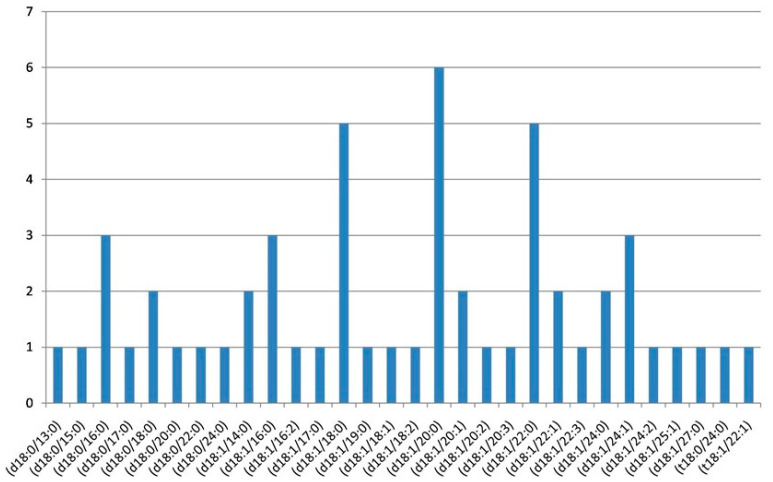
Plot of the number of gangliosides species in cavernous hemangioma vs. ceramide compositions. Reprinted with permission from [67].

In the context of human brain hemangioma, another instance of a ganglioside exhibiting the potential to serve as a biomarker was identified by MS. This specific structure, occurring in the mass spectrum as a signal of moderate intensity at the *m*/*z* 1031.6579, was confidently assigned with a mass accuracy of 4.7 parts per million (ppm), to the monodeprotonated form of *O*-Ac-GM4 (d18:1/16:0). Another important aspect is that HR MS revealed for the first time the high variability in fatty acid compositions in the ceramide moieties of gangliosides expressed in hemangioma, from species presenting long chains which encompass from 22 to 27 carbon atoms, to structures of shorter chains, usually between C13 and C18 fatty acids. The histogram in Figure 1 presents the distribution of the native ganglioside species in cavernous hemangioma, classified according to ceramide [67].

Recognizing the distinctive features of cavernous hemangioma, which are marked by gangliosides exhibiting a short carbohydrate chains and a limited number of Neu5Ac moieties integrated into the primary glycan structure, a structural CID analysis was conducted on the precursor ion identified at an observed mass-to-charge ratio (*m*/*z*_exp_) of 775.95122 in the mass spectrum. This ion was associated with the double deprotonated GD3 (d18:1/24:1) species [67]. The precursor ion was isolated within 1 *m*/*z* unit window and then subjected to CID at collision energies ranging from 30 to 65 eV, varied during the experiment. In an effort to elucidate the structure of the ceramide, important information was obtained from the U^−^ and T^−^ ions observed at *m*/*z* 365.3418 and *m*/*z* 390.3433, respectively. These ions provided a crucial insight into the fatty acid chain composition, specifically featuring 24 carbon atoms along with a double bond. This detail offers valuable understanding of the fatty acid component within the ceramide. At the same time, the Q^−^ fragment ion, detected at *m*/*z* 265.1474, stands out. This ion was of major importance as it served to confirm the presence of the sphingoid base composition (d18:1) in the ceramide structure, clarifying a fundamental aspect of its chemical composition [67].

HR MS and MS/MS utilizing CID provided a more detailed representation and a deeper understanding of the expression and complex structure of sialylated glycolipids in the context of human cerebral hemangioma. These advanced techniques have paved the way to revealing the fine details of these glycosidic compounds, offering a clearer picture of how these glycoconjugates influence and participate in the specific biological processes associated with this condition.

Another benign brain tumor with a high incidence is meningioma, first described in 1614 by Paster [70], as a primary brain benign tumor of the CNS [71], which arises from the protective membranes of the brain and the spinal cords, i.e., the meninges [72]. According to Central Brain Tumor Registry of the United States (CBTRUS), between 2016 and 2020 meningioma accounted for 40.8% of all tumors and 56.2% of all benign tumors. The meningiomas have the highest incidence rate of all types of benign tumors, 9.64 per 100,000 population, with an incidence rate higher in females (13.56 per 100,000) than in males (6.14 per 100,000) [73]. These tumors can elevate intracranial or spinal cord pressure by either stimulating or damaging nearby nerve tissue, thus displacing the mass within a confined space, leading to the manifestation of symptoms [71], which can be influenced by factors such as the tumor’s size, its specific location, and its proximity to essential structures within the body. Typical signs may encompass ongoing headaches, epileptic episodes, visual issues such as blurred or reduced peripheral vision, hearing impairments or tinnitus, limb weakness or numbness, challenges related to balance and coordination, alterations in cognitive abilities or personality, as well as disruptions in hormonal equilibrium [72]. Meningioma can be diagnosed based on the symptomatology under a clinical neurologic examination, using imaging techniques such as CT, MRI, digital subtraction angiography (DSA), magnetic resonance spectroscopy (MRS) [74], cerebral angiography (CAG), single photon emission computed tomography (SPECT) and positron emission tomography (PET) [75]. Some radiological characteristics of meningioma are: (i) the irregular shape of tumor; (ii) the tumor volume which grows faster than 3 cm^3^/year; and (iii) the high blood flow in the surrounding area [76]. The most common treatments for meningioma are microneurosurgery, image guided surgery, radiotherapy and stereotactic radiotherapy in case of optic nerve meningiomas. Additionally, hormonal therapy, chemotherapy, antiangiogenic therapy [74] and endoscopy [75] are employed as treatments.

World Health Organization (WHO) classified meningioma according to the histopathology and molecular features in 15 different subtypes, which are separated into three tumor grades [77] based on brain invasion, necrosis, type of cell, mitotic activity and cellularity. Grade I meningiomas represent benign meningiomas, grade II the atypical meningiomas and grade III the anaplastic meningiomas [75]. While meningiomas are typically benign, tumors of higher grades exhibit a propensity to advance and reoccur [78]. Consequently, for a more reliable diagnostic of meningioma subtypes and their degree, as well as for a more effective treatment, a series of studies were carried out over the years in order to identify biomarkers associated with meningioma. The types of biomarkers analyzed were genetic, proteomic and glycomic. Meningiomas occur either spontaneously, in patients who suffer from neurofibromatosis type 2 (NF2), Li-Fraumeni (*TP53/CHEK2*), Turcot, Gardener, von Hippel-Lindau (VHL), Cowden (*PTEN*), Gorlin (*PTCH1, SUFU*), Werner (*LMNA*) or multiple endocrine neoplasia type I (*MEN1*) sindroms [75,79]. The most frequent gene mutation was found in *NF2* located in chromosome 22q. Moreover, a loss of heterozygosity (LOH) [77,78,80,81,82] was found in this chromosome. Some genetic alterations discovered in higher grade meningioma are deletions on 1p, 6q, 10q, 14q, 9p (*CDKNA*, *p14^ARF^*, *CDKN2B*) and 18q chromosomes and gains on 1q, 9q, 12q, 15q, 17q, and 20q [77,78,79,80,82]. Several genes associated with oncogenesis of meningioma are: *TRAF7, ATK1, KLF4, SMO, PIK3CA, BAP1, POLR2A, SMARCB1, AKT1E17K, hTERT*/telomerase, *MADH2, MADH4, APM-1, DCC, CDKN2A, p14^ARF^, CDKN2B, TP53, MEG3, ALPL, Notch, WNT, IGF, NDRG2, TERT, H3K27me3, Cx43*, *SMARCE1, AKP12, ARID4B*, DNA methylation and loss of heterozygosity of DAL1 [76,77,79,80,81,82,83,84,85,86,87].

Proteomic analysis has revealed alterations in protein levels downstream from meningiomas, and these changes are associated with specific tumor spatial patterns. Proteomic studies found an overexpression of PDZ and LIM protein 2 (*PDLIM2*/mystique/SLIM) and multiple proteins in meningioma samples, including serpin peptidase inhibitor alpha 1, ceruloplasmin, hemopexin, albumin, C3, apolipoprotein, haptoglobin, amyloid P-component serum, alpha-1-beta-glycoprotein, alpha-2-macroglobulin, and antithrombin-III. RB1 S780 was also found to be significantly higher [83]. Some proteins reported in other studies are integrin, WNT, RAS, FGF, EGF in tissue samples, an increase in APO E, APO J, A1AT proteins and a decrease in PTGDS, TTR, B2M proteins in cerebrospinal fluid and vimentin, alpha-2-macroglobulin, APO B, APO A-I and antithrombin-III in serum. In addition, *SERPINA1*, *CP, HPX, APOA1, ALB, C3, A1BG, HP* and *APCS*, were found in tissue, cerebrospinal fluid and serum samples [68]. In addition, other blood markers were found, such as serum *TIMP1/2*, *HER2* and plasma Fibulin-2 [76].

Meningioma gangliosides began to be studied in 1965 and for more than 20 years GM3 and GD3 species were the sole species considered associated with this tumor [88]. In 1991, the relation between partial/total loss of chromosome 22 with the expression of gangliosides in meningioma [89] was reported. The study revealed that GM3 was predominant in meningiomas without monosomy 22 and GD3 in meningioma with a total loss of this chromosome. In addition, in this work, GM2, GM1, GD1a, GD1b and GT gangliosides were found to be markers of meningioma. The distribution of GM1 and GD1a was lower in meningiomas with monosomy 22 and the distribution of GD1b and GT was increased in comparison with the meningiomas without monosomy 22. Almost two decades later, serum gangliosides were investigated in meningioma for the first time in order to differentiate their concentration before and after surgery [90]. The results demonstrated that: (i) the production and release or shedding of gangliosides by tumor tissue determine the concentration of total gangliosides in serum of cancer patients; (ii) the proportion of GD3 ganglioside decreased after surgery, while the GM3 proportion increased after surgery.

**Figure 2:**
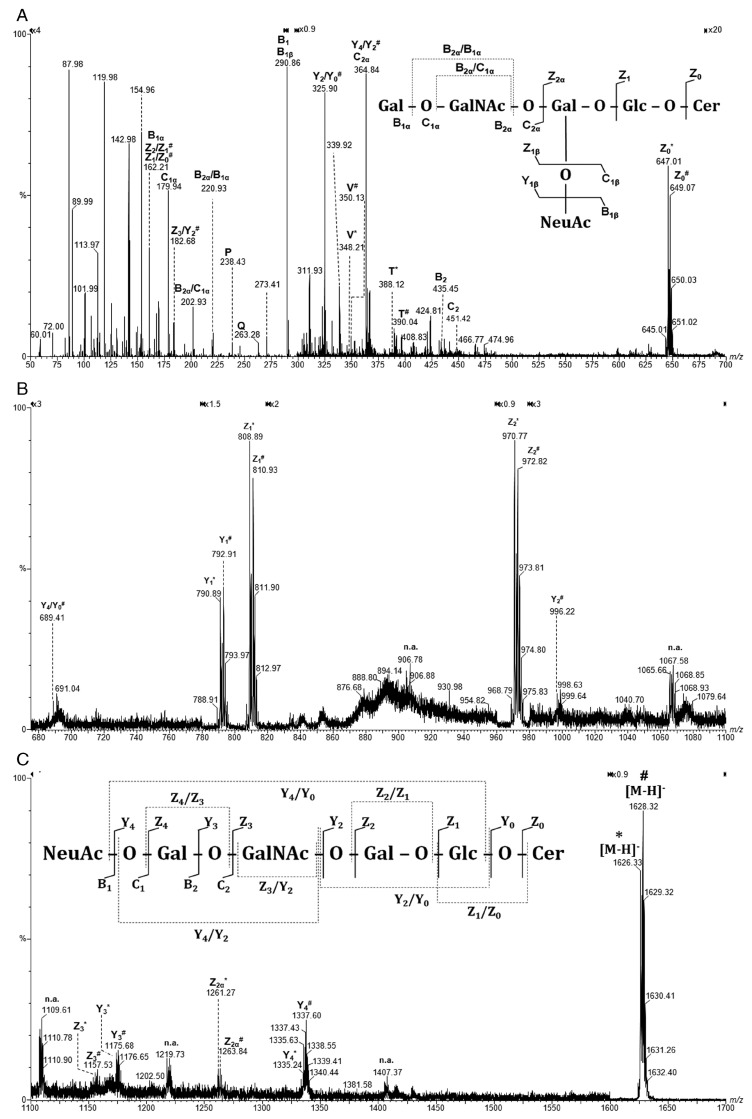
Fully automated (-)nanoESIchip-QTOF CID MS/MS of the [M-H]^−^ ions at *m*/*z* 1626.23 and *m*/*z* 1628.22 detected in the ganglioside mixture from angioblastic meningioma, isolated and fragmented simultaneously. (**A**) *m*/*z* (60–660); (**B**) *m*/*z* (680–1100); (**C**) *m*/*z* (1100–1700). Insets: Fragmentation schemes under the employed CID conditions of GM1b (in A) and GM1a (in C) isomers. Acquisition time 2 min; collision energy within 30–80 eV range (E_lab_). Assignment of the glycan and ceramide fragment ions is according to the published nomenclature [92,93]. Y* Z* and V* fragment ions diagnostic for GM1 (d18:1/24:1). Y^#^ Z^#^ and V^#^ fragment ions diagnostic for GM1 (d18:1/24:0); n.a., not assigned. Reprinted with permission from [91].

**Figure 3:**
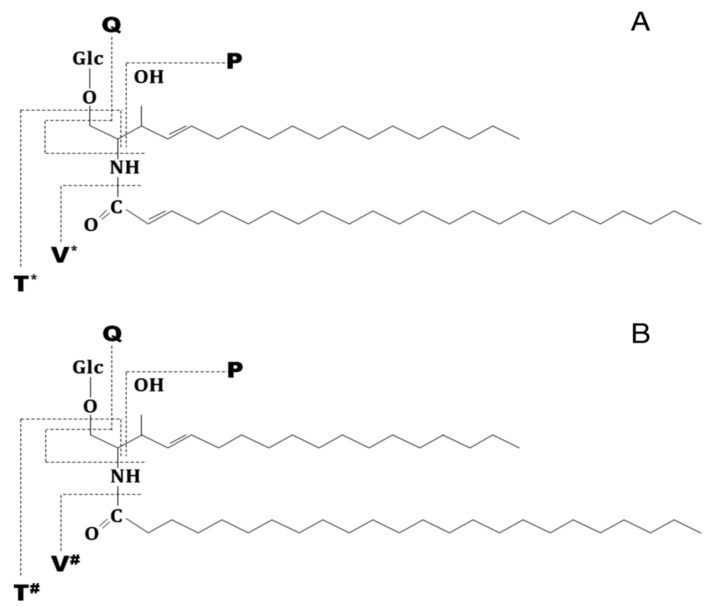
Fragmentation schemes under the employed CID conditions of: (**A**) Cer (d18:1/24:1) and (**B**) Cer (d18:1/24:0). Assignment of the ceramide fragment ions is according to the published nomenclature; V* and T* fragment ions diagnostic for GM1 (d18:1/24:1). V^#^ and T^#^ fragment ions diagnostic for GM1 (d18:1/24:0). Reprinted with permission from [91].

The first MS application on meningioma gangliosides was reported by our group in 2012 and targeted: (i) mapping and sequencing of the ganglioside from meningioma using a hybrid quadrupole time-of-flight (QTOF) mass spectrometer coupled to the fully automated nanoESI chip on the NanoMate robot; (ii) quantification of meningioma gangliosides by HPTLC and laser densitometry and (iii) detailed structural characterization by fragmentation analysis using CID MS/MS of the species with biomarker role or clearly associated with meningioma [91]. The gangliosides from meningioma tissue were extracted and purified from a 54 years old male. The densitometric analysis of the distribution of gangliosides in meningioma compared to healthy cerebellar tissue revealed, first of all, that GM3 and GD1a fractions were 48.8% and 34.8%, respectively, of the total ganglioside content, and that the distribution of GM3 and GD1a was higher in meningioma tissue, while the distribution of GM1, GM2, GD3, GD1b, and GT1b was lower. The MS screening revealed an unexpectedly rich molecular ion pattern corresponding to no less than 34 ganglioside components of short glycan chains, mono- or disialylated, with the highest expression of GM3 and GM1. GM4 and five asialo (four LacCer and one GA2 species), which were not recognized by HPTLC, were also discovered by chip-based nanoESI MS. In terms of GM1 structures, ions corresponding to nine glycoforms were detected, of which the signals at *m*/*z* 1626.23 and 1628.22 assigned to GM1 (d18:1/24:1) and/or GM1 (d18:0/24:2) and GM1 (d18:1/24:0), respectively, were the most abundant. Consequently these ions were isolated and submitted to fragmentation analysis by CID MS/MS (Figure 2 and Figure 3) which confirmed both ceramide structures (Figure 3) and the localization of Neu5Ac at the outer and, respectively, inner Gal, a feature consistent with GM1a and GM1b isomers, as biomarkers of human meningioma (Figure 2).

### 2.2. Malignant Tumors

Gliomas, as the name implies, arise from glial cells and are the most prevalent and aggressive primary tumors of the CNS [94], comprising nearly 80% of all brain malignancies [95,96,97]. Historically, gliomas were diagnosed and classified according to the (i) malignancy grade, from grade I to grade IV based on the degree of proliferation indicated by the mitotic index and the presence or absence of necrosis [85,98]; (ii) cell type, including astrocytomas, ependymomas, and oligodendrogliomas [94,99] and (iii) location, whether they are above or below the tentorium [100].

The last several decades of research invested into the molecular profiling of gliomas, as well as the multi-institutional collaborations on the topic have significantly advanced our understanding of these clinically and molecularly heterogeneous neoplasms, and provided new insights into tumor initiation, ontogeny, and progression [101,102]. Hence, the latest discoveries in the field simultaneously with the updated WHO 2016 classification of gliomas [98,103], which includes also the molecular and genetic diagnostic criteria, have a significant impact on the diagnosis and management of many different subtypes of gliomas, including a more specific prognostic and therapeutic benefits for patients with gliomas [96,99,102].

Astrocytomas (AcTs), the most widespread gliomas, develop in the CNS and originate from astrocytes, a type of star-shaped glial cells in the cerebrum. Depending on how fast the AcTs are growing and the likelihood that they will spread to nearby brain tissue, WHO classified astrocytomas in benign (noncancerous) or malignant (cancerous) neoplasms [85,102,104]. WHO grade I, known as pilocytic astrocytoma are often benign, slow-growing tumors, usually encapsulated, preserve clear borders between normal and tumor cells, are localized most often in the cerebellum, are largely cured (96% survival rate at 5 years), and can be resected by surgery. Grade II, known as diffuse astrocytoma, infiltrative or low-grade gliomas, are relatively slow-growing invasive tumors with poorly defined borders, which will acquire a more aggressive phenotype over time. They may progress to glioblastoma, and cannot be entirely separated from the surrounding brain during surgery and tend to recur after treatment. WHO grade III, known as anaplastic astrocytomas grow faster and more aggressively than grade II AcTs, are more common in men than women and include a larger brain invasion, for which radiation and chemotherapy are required following surgery. The most advanced form, WHO grade IV, known as glioblastoma (GBM), is also the most common (60%), malignant, invasive, aggressive and deadliest type of AcTs, with a median survival of about 1 year [105]. GBM is characterized by a fast-growing phenotype with the presence of necrotic regions and vascular development [96]. Primary GBMs develop de novo, whereas secondary GBMs originate from lower grade glioma. They can either begin as a grade 4 tumor—primary GBM—(90% of cases), or present as a cancerous progression from a previously existing lower-grade AcT—primary GBM—(10% of cases).

Significant dissimilarities were observed among the four degrees of malignancy: (i) the incidence increases with the degree of severity (grade I AcT accounts for 2%, while GBM 24% of all brain tumors); (ii) age plays an important role, since the older the patient, the higher the chance that AcT will be of a higher grade, except for grade I which is most common in the pediatric population [99]; (iii) men have a higher risk of developing grade III and IV gliomas compared to women, an aspect that might be related to hormonal factors and genetic features [94]; and (iv) low grade AcT tend to be of larger size prior to becoming symptomatic, as compared to higher grade AcT.

Affecting nearly all parts of the brain, sometimes even the spinal cord, AcTs can lead to up to the destruction of neural tissue. Since gliomas, in general, and AcT, in particular, represent a regular cause of mortality and morbidity in both the young and elderly, in order to avoid the high morbidity and mortality associated with this condition, a prompt diagnosis and treatment is mandatory [106]. The imaging tests, particularly MRI with administered contrast, which is the most sensitive test available to diagnose malignant AcT, or CT scan, are currently the gold diagnostic procedures, since these techniques can aid physicians to determine the size and location of an AcT, and further, to recommend an appropriate treatment approach. All advances in imaging technology have improved the accuracy of pre-operative diagnosis and the median survival rate at 5 years from about 40–50% (which was specific prior MRI era) up to 65% and at 10 years from 20–30% up to 40% [107].

However, since (i) the incidence of AcT has increased annually by 1–2% in the past years [108]; (ii) many glioma patients present non-specific symptoms such as headaches [100]; (iii) AcT is not diagnosed until patients have progressed to the symptomatic phase which drastically decreases the chances of survival and also minimizes treatment efficiency; and (iv) MRI or CT tests involve long waiting time and high costs [109,110], the current trend is represented by prediction and precise early diagnosis based on biomarker discovery, including genes, proteins, lipids and other molecules unique to the tumor [111,112,113,114,115,116,117,118,119,120,121,122,123], prior the clinical symptoms to arise, at a stage when the resection is possible.

Currently, little is known about pre-diagnostic biomarkers that predate glioma detection that could improve the earlier detection. In order to overcome this issue, Andrews et al. [109] provided in 2023 an update related to the evidence in the literature for pre-diagnostic biomarkers in glioma, including the grow factors, metabolomics and proteomics. Moreover, in 2022 Ran et al. [124] manually extracted accurate information on 406 glioma diagnostic biomarkers from 1559 publications (from May 1989 to May 2022), including biomarker descriptions, clinical information, associated literature, experimental records, associated diseases, statistical indicator and conceived GlioMarker, the first thorough and comprehensive database for knowledge exploration of glioma diagnostic biomarkers.

Since aberrant cell-surface glycosylation patterns were found to be characteristic for all tumors and were linked to tumor progression, metastasis, and invasiveness, although the precise molecular mechanisms are poorly understood, gangliosides still represent valuable diagnostic markers of malignant CNS tumors. The first assessments of ganglioside composition in human AcT were reported in the late 1970s and early 1980s and revealed a decreased percentage of ganglioside content not only in AcT but also in adjacent tissue [125] compared to healthy tissue [125,126], especially GD1b, GT1b [125], and a significant increase in GM3 [126]. These findings suggest that AcT tumors shed sialoglycolipids into the circulation, an aspect with significant biological consequences that was further confirmed in cerebrospinal fluid (CSF) as well [127]. By using immunostaining with monoclonal antibody R24 and enhanced chemiluminescence detection, Ladisch et al. [118] reported a significantly higher GD3 level in patients with medulloblastomas (*n* = 9) and AcT (*n* = 10) than those of controls (mean ± SD 44.7 ± 8.4 *versus* 18.2 ± 1.9 pmol/mL, *n* = 20, *p* < 0.0002), while their MS analysis showed a heterogeneous ceramide structure for ganglioside GD3 in tumors, and a selective shedding of species with shorter fatty acyl chains.

Over the years, the published studies comparing the ganglioside profile in AcT with other primary brain tumors, or healthy brain tissue, using different classical approaches, such as high affinity anti-ganglioside antibodies using immunohistochemistry [128], HPTLC coupled with densitometry [129], two-dimensional TLC [130], and HPLC [131], confirmed the simplification of ganglioside composition in pilocytic astrocytomas grade I. However, all these studies aimed only at the quantitative analysis of gangliosides, and not the compositional analysis from the ceramide point of view.

To our knowledge, there is only one thorough study on the characterization, mapping and structural elucidation of gangliosides with potential biomarker values in a low-grade AcT. This study, carried out by our group, represents a comparative assay on gangliosides extracted and purified from AcT, its surrounding tissue (ST) and a normal control brain tissue (NT) under identical conditions. Performed using HR MS on an Orbitrap instrument [132], our research disclosed a distinct ganglioside pattern in AcT and ST compared to NT. The employed high resolving power and mass accuracy allowed the detection and identification of a large number of species in the three samples, namely 37 in AcT, 40 in ST and 56 in NT. The comparative overview on the ganglioside structures detected in AcT, ST, and NT presented in Table 2, Table 3 and Table 4 reveals several other valuable findings: (i) AcT and ST contained 18 identical components, while NT only one common structure with ST and two with AcT; (ii) AcT was characterized by a higher sialylation degree (32 polysialylated glycoform), while in NT GM-type of species (18 structures) prevail; (iii) the number of species with more than two Neu5Ac moieties was higher in ST compared to AcT and NT; and (iv) the concept of tumoral cell protrusion in ST was supported by the *O*-acetylation and *O*-fucosylation of gangliosides, which were found higher in AcT and ST compared to NT [131]. Considering that in AcT hypersialylation might be responsible for the development of AcT cells [133], the elevated degree of sialylation characteristic for ST confirmed as well the infiltration of AcT cells in the surrounding tissue. Moreover, the incidence of ceramides with long chain fatty acids (LCBs), exceeding 25 carbon atoms was also associated with AcT and ST. These preliminary data might suggest a possible association of such structures with tumor development and invasion. Carbohydrate sequence analysis in MS^2^–MS^4^ experiments on a GT1 (d18:1/18:0) or GT1 (d18:0/18:1) species identified in all three samples completed and supported all the findings from the MS assay, since it highlighted the incidence of GT1c isomer in AcT and ST, but not in NT [132].

Considering the elevated frequency, aggression, morbidity and mortality of GBM, the main research direction of primary brain tumors was focused on GBM and oriented towards the (i) evaluation of the molecular mechanisms related to the tumor aggressiveness; (ii) identification of new methods to prevent invasiveness; (iii) discovery of more effective therapeutic schemes; and (iv) identification of pre-diagnosis biomarkers [134].

With a reduced abundance in the healthy adult human brain and an increased expression in various malignant cancers, including gliomas, GD2/GD3 gangliosides were reported over the time to be tumor-associated antigens [135,136,137,138] and potential targets for anti-tumor vaccination therapy [61,139,140,141]. Therefore, of major importance in deciphering the role of GD3/GD2 in the GBM tumor cell proliferation and invasion is the achievement of a precise mapping of ganglioside expressed in the aberrant tumor tissue.

The ganglioside content and composition, especially of lb gangliosides, in different types of glioma tumors, including anaplastic astrocytomas, GBM and gliosarcoma, were correlated to malignancy grade and median survival time [142]. The investigation of von Holst [143] on glioma-associated gangliosides in biopsies from 44 patients with astrocytoma grade II, anaplastic astrocytoma, anaplastic oligodendrogliomas, and GBM, evidenced a strong expression of GM3 and GD3, which support their involvement in proliferation, and dedifferentiation of high malignancy grade tumors [143]. In an ample investigation based on HPTLC and laser densitometry, Radić et al. [90] assessed the differences in serum gangliosides content and composition one week before and one week after the surgical removal of different brain tumors, in order to estimate a potential prognostic value of these differences. An important aspect observed by the authors is that the complete or partial tumor removal influenced the trend of postoperative ganglioside concentration. Hence, in the case of partial tumor removal (glioblastoma, astrocytoma, oligodendroglioma), the concentration of serum gangliosides increased postoperatively, while in the case of completely removed tumors, a postoperative decrease in GD3 and an increase in GM3 proportion were observed [90]. More recently, a comparative and comprehensive structural and compositional characterization of gangliosides in GBM, corresponding peritumoral tissue and healthy brain tissue, to disclose their roles as tumor-associated antigens was conducted by MS and HPTLC [144]. The qualitative and quantitative characterization by HPTLC evidenced a five times lower total ganglioside content in GBM compared to healthy brain tissue. GD3, which accounted for 53% of the total ganglioside content, together with GM3, GM2, GD2 and *O*-Ac-GD3/nLM1 fractions were more highly expressed in GBM than in the peritumoral and normal brain tissue. Moreover, the proportions of more complex species, such as GM1, GD1a, GD1b, GT1b, which are characteristic of adult human brain, were considerably lower in GBM compared with the normal brain and peritumoral tissue [144]. Further, the compositional analysis by MS also revealed differences in the ceramide composition: ceramides with fatty acid chains from C16 up to C24, including C24:1 or the unusual C17, C19 were detected and associated with GBM, while normal and peritumoral tissue contained mainly ceramides with C18. Although the composition of main species in peritumoral tissue reflects the normal brain ganglioside pattern, the higher abundance of GM1, nLM1/GM1, GM3 and GD3 detected in peritumoral tissue, points out that important processes between tumor and its environment occur within this area. Fabris et al. [144] also reported differences in the *O*-acetylation profile; while *O*-Ac-GD1 was detected in only normal and peritumoral tissue, *O*-Ac-GD3 species were found solely in GBM. Additionally, the CID MS/MS experiments structurally validated a novel GBM associated *O*-Ac-GD3 isomer, for which the *O*-acetylation was linked to the inner sialic acid residue. Previously, such an isomer was detected in a gliosarcoma specimen [136].

**Scheme 2:**
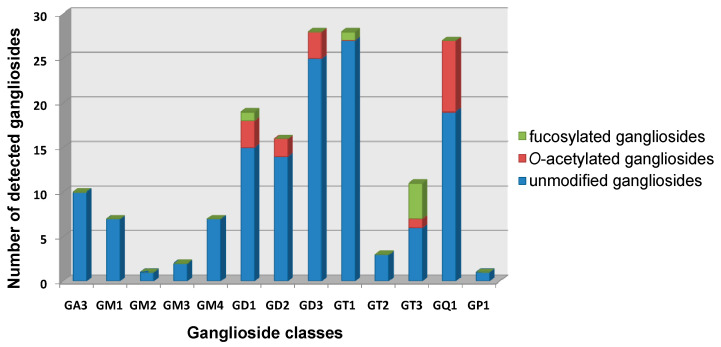
Chart plotting the number of the identified ganglioside species in GBM according to the composition of their glycan core and the sialylation degree. Reprinted with permission from [148].

Considering the constant need for development of more powerful and sensitive methods that are capable of discriminating and identifying low abundant gangliosides in complex mixtures, and the demonstrated potential of IMS MS in this direction [44,48,145,146,147], in 2021, we reported the first investigation of GBM-specific gangliosides using nanoESI IMS MS [148]. NanoESI IMS MS in negative ion mode, revealed a clear distribution of the chemical noise across a wide range of drift times and the ganglioside separation into mobility families according to their charge state, carbohydrate chain length and the degree of sialylation [148]. Such an IMS MS separation pattern was previously reported by us and others as specific to this type of molecules [44,48,145,146,147]. Unlike the experiments with direct infusion, which generate a total ion chromatogram, of major importance when investigating IMS MS samples as GMB, where the concentration of gangliosides is considerably reduced compared with healthy brain tissue, is the possibility to extract the data from DriftScope on small regions of interest. Hence, the drift time retention of narrow areas led to the identification of 160 different molecules [148], a number of ganglioside structures three times higher than identified before in GBM with no separation prior to MS [136]. Inspection of Scheme 2 revealed an elevated structural diversity, with the predominance of GD3 and GT1 glycoforms, with 36% GD3 and 36% GT1 of the total number of discovered gangliosides. The superior sensitivity of the instrument and the option to integrate the data over narrow regions also permitted the detection and identification of (i) unusual glycoforms, such as GM4, GM3 and GA3 specimens, characterized by short a glycan chain and reduced sialic acid content; (ii) highly sialylated gangliosides, including tetra- and pentasialylated glycoforms; (iii) *O*-acetylated and *O*-fucosylated structures; (iv) structures with fatty acid chains from C12 to C28; (v) gangliosides characterized with preponderance by unsaturated and polyunsaturated fatty acids residues; and (vi) species containing ceramides with an odd number of carbon atoms, from C17 to C23, mainly with C17 and C19 [148], a feature also observed by Fabris et al. [144].

While there are plenty of studies demonstrating GD3 involvement in brain development, and its markedly increased expression in cancers, less is known about its implication in GBM of GT1 forms. Although Hamasaki et al. [149] proposed GT1b isomer as a brain metastasis-associated glycoform, this species was never related with GBM. In view of these findings, the structural investigation performed in the transfer cell, of a trisialylated species bearing (d18:1/24:1) documented through the generated diagnostic fragment ions and the incidence of a single mobility feature, the results documented the incidence of the GT1c isomer in GBM [148].

The first insight into the histology-specific accumulation of different lipids, including gangliosides, involved in GBM cell metabolism and signaling, was recently reported by O’Neill et al. [150]. The high-resolution MALDI MS imaging revealed a differential accumulation in tumor and endothelial cell subpopulations of gangliosides, including the glioblastoma stem cell marker, GD3, correlated with their fatty acid residue composition [150]. A similar approach, based on MALDI MSI applied by Ermini et al. [151] for the investigation of ganglioside metabolism and distribution, was able to distinguish between rat intracranial allografts of rat gliomas and human medulloblastoma. Hence, MALDI MSI revealed a series of differences: (i) medulloblastoma xenograft expresses GM2, and lacks GM3, healthy adult brain lacks in GM3, while glioma allografts expresses GM3; (ii) in the healthy adult rodent brain, GM1 and GD1 were the main types of glycolipids; (iii) GM3 (d18:1/24:0) was identified as the most abundant ganglioside species in the glioma allotransplant; and (iv) mouse xenografts of human medulloblastoma were characterized by prominent expression of the GM2 (d18:0/C18:0) [151].

Considering that, besides the blood-brain barrier and the tumor-propagating microenvironment, the incidence of cancer stem cells (CSC) is responsible for the resistance of GBM to treatment, and that GBM CSC expresses glycolipids recognized by the A2B5 antibody, Baeza-Kallee et al. [152] studied the effect of neuraminidase administration at the cellular level to tackle human GBM CSC. Their ample investigation based on flow cytometry, DNA methylation transcriptomic analysis, real-time Quantitative PCR analysis and LC-MS on an Aquity UPLC H-Class PLUS system and a triple quadrupole (QqQ) MS, demonstrated that neuraminidase (i) decreased A2B5 expression, tumor size and regrowth after surgical removal in the organotypic slice model; (ii) did not induce a distinct transcriptomic or epigenetic signature in GBM CSC lines; (iii) drastically reduced ganglioside expression in GBM CSC lines and (iv) by its pleiotropic action, is an attractive local treatment against GBM.

The analysis by Chahlavi et al. [153] of individual HPLC fractions from two apoptogenic GBM lines (CCF52 and U87) revealed that CD70 and gangliosides are both synthesized by GBMs that may be key mediators of T-cell apoptosis and may contribute to the T-cell dysfunction noticed within the tumor microenvironment. The HPLC and MS investigation showed that all four apoptogenic GBM lines abundantly synthesized GM2, GM2-like gangliosides and GD1a, while in the case of the two GBMs lacking activity, there was no such synthesis. Moreover, gangliosides isolated from GBM lines as well as HPLC fractions containing GM2 and GD1a were directly apoptogenic for T cells [153].

A rare form, with only about 200 case reports, of high-grade glioma that has both glioblastoma and sarcoma components and is more prone to extracranial metastasis than other gliomas, is gliosarcoma [106]. Although it is an extremely rare neoplasm, over the years several attempts to characterize gliosarcoma, either alone, or in comparison with GBM were undertaken [154,155,156,157,158,159,160]. As with GBM, the poor prognosis with a median survival of about nine months, and the failure of gliosarcoma treatment, are due to the tumor aggressiveness, which causes extensive infiltration of the tumoral cells into the surrounding healthy brain tissue. In order to target the invading tumor cells by using specific binding ligands [161], one of the investigated treatment strategies, the identification and detailed characterization of tumor-specific target molecules, is therefore mandatory.

**Figure 4:**
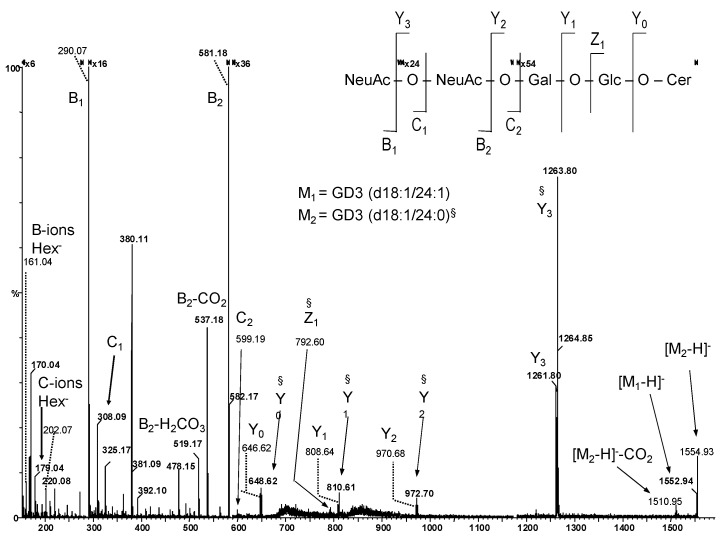
Negative ion mode nano-ESI-QTOF MS/MS of the [M-H]^2−^ ions at *m*/*z* 1552.94 and *m*/*z* 1554.93 corresponding to the GD3 (d18:1/24:1) and GD3 (d18:1/24:0), respectively, detected in gliosarcoma sample. Solvent, methanol; ESI voltage; 1000–1250 V; for precursor ions isolation the LM and HM parameters were set to 3; collision energy; 30–45 eV; collision gas pressure: 15 psi; acquisition: 3 min; average sample consumption: 1 pmol. Inset, the fragmentation scheme of GD3. Reprinted with permission from [136].

In view of the known ganglioside implication in malignant transformation and tumor progression/invasiveness, a systematic characterization of ganglioside composition in human gliosarcoma *vs*. healthy brain tissue employing HPTLC complemented by two high-resolution MS strategy, was reported by our groups [136]. Important difference between the two samples were observed from the first stage of research conducted by HPTLC. The quantification data revealed that the ganglioside content in gliosarcoma is 7.4 times lower than in the normal brain tissue used as a control. Chip-based nanoESI QTOF MS and nanoESI FTICR MS screening, in excellent agreement with HPTLC pattern, documented as well the highly altered ganglioside expression in gliosarcoma. The relative abundances of GD3, GD2/GT3, and GM3 fractions higher, whereas GM1/nLM1, GD1/nLD1, and GD1b fractions lower relative abundance. In total, over 70 distinct glycoforms, a considerably higher number than previously reported in other gliomas specimens, were detected and identified in gliosarcoma [136]. MS also revealed a prominent abundance of *O*-Ac-GD3 (d18:1/18:0) and (d18:1/20:0), and supported the presence of some unusual minor species, such as GM4, Hex-HexNAc-nLM1, Gal-GD1, GT3, Fuc-GT1, GalNAc-GT1, *O*-Ac-GM3, di-*O*-Ac-GD3, and *O*-Ac-GT3, not previously reported as glioma-associated gangliosides. Additionally, an over expression of GM2, GM1, and GD1 species along with a poorly expression of GT1 was also found to be characteristic for gliosarcoma [136]. NanoESI QTOF CID MS/MS was further used to validate the species highly abundant in gliosarcoma as well as those recognized as far as brain tumor-associated antigens. For example, the product ion spectrum illustrated in Figure 4, documented through the diagnostic fragment ions, the structure of two such glycoforms, characterized by short glycan chain and unusual ceramide composition, namely GD3 (d18:1/24:1) and GD3 (d18:1/24:0) [136].

Several main achievements of MS are emphasized in relation to gangliosides in primary malignant brain tumors: (i) a variety of novel glycoforms were detected, identified and added to the currently existing panel of AcT and glioma tissue-associated structures; (ii) novel potential biomarkers, suitable for clinical applications were discovered and completely characterized in tandem MS by CID; (iii) investigation of ganglioside biomarkers in biological fluids can detect and diagnose the disease in early phases, highlight the risk of disease development and progression, and provide accurate information about the patient’s response to a certain treatment; and (iv) each MS methodology and approach, especially when combined with separation techniques, may provide crucial structural data, relevant to ganglioside roles in brain tumor biology, differential diagnosis/prognosis as well as personalized and targeted treatment.

## 3. Gangliosides in Brain Metastases

Among intracranial tumors, brain metastases represent the forms of cancer with the highest incidence. Since the efficiency of the modern therapeutic schemes for cancer improved to a large extent the overall survival rate, the number of oncologic patients living much longer with the systemic disease and reaching the stage of brain metastases has increased significantly [162]. Lung adenocarcinoma is the most frequent type of primary malignant tumor which spreads to the brain [163] to give metastasis as the main complication, being followed by breast cancer, melanoma, colorectal cancer and renal carcinoma. Currently, brain metastases are diagnosed on the basis of the imagistic methods, in particular MRI and, depending on the localization and symptoms, treated by surgery, stereotactic radiosurgery, whole brain radiation therapy, chemotherapy and immunotherapy [163,164]. In the terminal stage, palliative medication is provided in order to improve the life quality and alleviate symptoms such as headache, nausea, neurological impairments and seizures. Similarly to the metastatic cancers in other organs, brain metastases retain all the histological features of the primary tumor from which they originate [162]; however, only the major molecular characteristics of them.

Being well-known biochemical markers of the CNS in health and disease, gangliosides were studied in brain metastases as potential therapeutic targets or for establishing diagnostic procedures based on molecular fingerprints. Several ganglioside species were found involved in the process of tumor cell proliferation and, ultimately, invasiveness into the cerebral tissue. Except for GT1b isomer, discovered almost 15 years ago [149] as a marker of brain tumors metastasized from colon, renal, lung, esophagus, pancreas and mammary carcinomas, recent studies suggest the implication of other ganglioside classes as well. For instance, using a nude mouse human xenograft melanoma brain metastasis model, Ramos et al. [165] discovered that the levels of GD3 gangliosides are extensively upregulated in melanoma brain metastasis. Likewise, the deficiency of GD3 synthase was found to attenuate glioma progression in a platelet-derived growth factor B-driven murine glioma model, which implies that GD3 also enhances the progression of glioma tumors [166]. In addition, GD3 and GD2 were revealed over- or differentially expressed in neuroblastoma, melanoma and triple-negative breast cancer, where they mediate cancer cell proliferation and migration to the brain as well as tumor angiogenesis [167,168,169,170]. On the other hand, some ganglioside structures were reported to slow down the metastatic process, among which monosialoganglioside, GM3, which was confirmed as an inhibitor of the angiogenesis in highly vascularized AcT [171].

Among all biochemical methods, mass spectrometry with either MALDI or microfluidics-based nanoESI provided one of the most comprehensive evaluation of the changes in ganglioside expression in the aberrant metastatic tissue. In 2009 [172] the gene *ST6GalNAc5,* was discovered over-expressed in breast cancer cells able to produce brain metastasis. Although at the time, the ability of human breast cancer cells to produce α-series gangliosides had not been demonstrated, the identification of *ST6GalNAc5* as one of the genes involved in breast cancer-derived brain metastasis raised the issue of the ability of breast carcinoma cells to synthesize α-series gangliosides. A few years later, by stable transfection, MS and MS/MS analysis of the total glycosphingolipid content Vandermeersch et al. [168] have shown that *ST6GalNAc5* expressing MDA-MB-231 epithelial-like breast cancer cell line, commonly used to model metastatic breast cancer, accumulate the GD1α ganglioside. The MS part of the study was conducted on a MALDI TOF/TOF instrument operating in the positive ion mode to detect and sequence the extracted glycosphingolipids, which were purified by reverse phase chromatography and permethylated prior to MS and MS/MS analysis. Via the following diagnostic sequence ions (i) B/Y-ion series documenting the terminal HexNAcHex (Neu5Ac)_2_ tetrasaccharide and excluding GD1a and GD1b isomers in which at least a Neu5Ac residue is linked to the internal galactose and (ii) the ion corresponding to HexNAcNeu5Ac disaccharide, MALDI-TOF/TOF fragmentation analysis by CID established the incidence of GD1α, and, consequently, that the expression of *ST6GalNAc5* cDNA in human cancer cells MDA-MB-231 results in the accumulation of GD1α.

**Figure 5:**
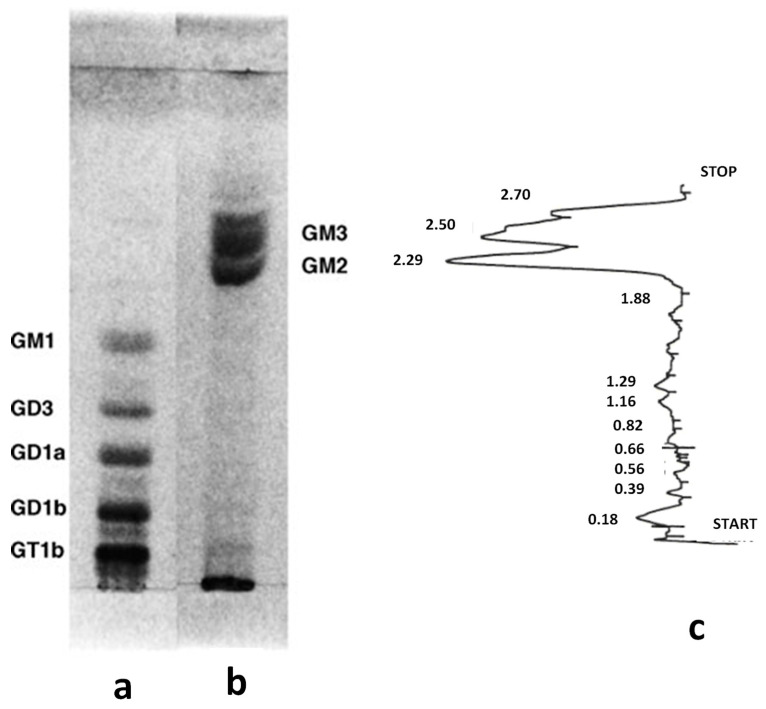
High performance thin-layer chromatography of gangliosides extracted from: (**a**) HB and (**b**) BMLA; (**c**) densitometric quantification of ganglioside fractions from BMLA. Reprinted with permission from [173].

Mass spectrometry was also involved by us in the analysis of the gangliosides expressed in brain metastasis of lung adenocarcinoma (BMLA) [173]. Chip-based nanoESI performed on the NanoMate robot was optimized for profiling and biomarker discovery in the native ganglioside mixture extracted and purified from BMLA diagnosed in a 73-y-old male patient. The measurements were conducted in the negative ion mode, on a high resolution instrument, i.e., a hybrid QTOF MS in laboratory coupled to the NanoMate robot via a specially designed mounting bracket. An age matched healthy brain (HB) tissue originating from the cerebellum of a subject deceased in a traffic accident was used as the control. Both BMLA and HB ganglioside mixtures were sampled and measured under identical solution and instrument conditions. The comparative HPTLC run in parallel for HB and BMLA as well as the ganglioside quantification by densitometric scanning (Figure 5) performed prior to chip-MS screening revealed that GM3, representing 52.27% of the total content, followed by GM2 with 34.81% correspond to the dominant ganglioside fractions of BMLA gangliosidome, whereas GD3 and the complex species GM1, GD1a, GD1b and GT1b exhibit a higher expression in HB [173].

**Figure 6:**
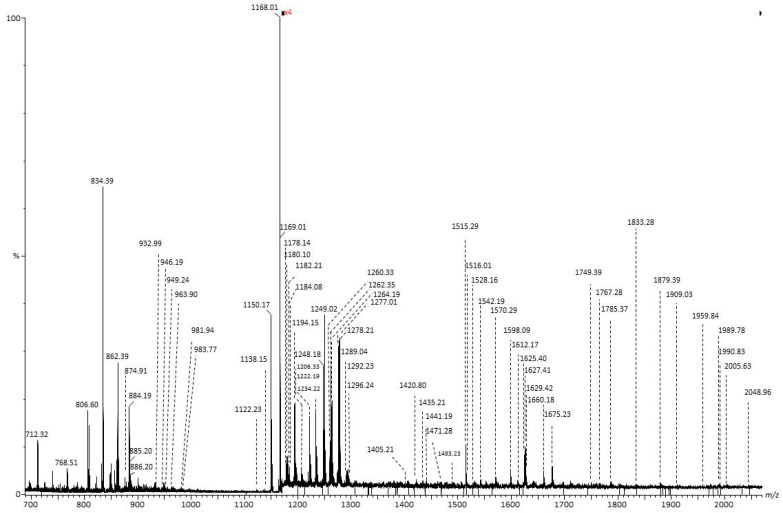
(-)Chip-nanoESI QTOF MS of the native ganglioside mixture extracted from BMLA. Solvent: MeOH; sample concentration 2.5 pmol/μL; acquisition time 1 min; Chip ESI: 1.5 kV; Cone voltage: 45 V. Reprinted with permission from [173].

Chip-based nanoESI MS screening in the negative ion mode and the follow-up comparative analysis revealed marked dissimilarities in the BLMA- (Table 5, Figure 6) and HB- associated gangliosidomes in terms of the number of occurring ganglioside species in the two tissues, the architecture of their glycan core, including the carbohydrate and non-carbohydrate types of modifications, and the structure of the ceramide moieties.

HB gangliosidome was discovered as highly complex, dominated by species containing different numbers of Neu5Ac moieties in the oligosaccharide chain. Hence, chip-based MS identified no less than 104 distinct signals corresponding to the entire series of mono- to hexasialo ganglioside species with or without peripheral attachments to the glycan core such as *O*-fucosyl or *O*-acetyl groups. The group of polysialylated gangliosides also included six tetrasialo GQ as well as the hexasialo GH2 (d18:0/24:1) and GH2 (d18:1/24:0), uniquely found in HB. The monsialo series encompassing GM1 (d18:1/18:0), GM1 (d18:0/18:1), GM1 (d18:1/20:0), GM1 (d18:0/20:1) and the fucoganglioside variant of the monsialotetraose, Fuc-GM1 (18:1/18:0), were found dominant in the spectrum, being identified as intense signals, which correspond to monocharged anions. The second category identified in the HB tissue through abundant ions is the disialo class, which encompasses 63 species among which GD1 (d18:1/18:0), GD1 (d18:0/18:1), GD1 (d18:1/20:0) and GD1 (d18:0/20:1). The third highly expressed category is represented by the rarely occurring fucogangliosides in the GM1 and GD1 class as well the 30 *O*-acetylated structures, including four di-*O*-Ac glycoforms. The *O*-acetylated species are of GM3, GM1, GD3, GD2, GD1, GT3, GT2, GT1 and GQ1 structure bearing ceramides of different constitutions.

**Figure 7:**
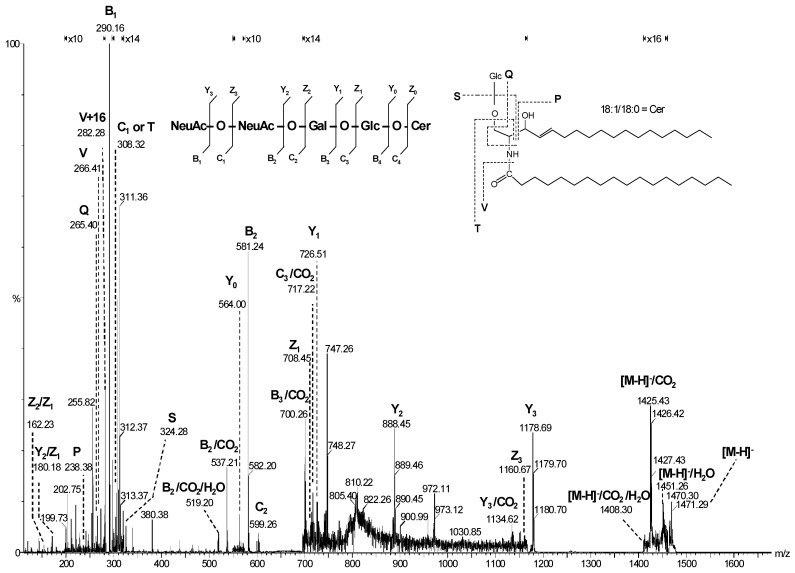
(-)Chip-nanoESI QTOF CID MS/MS of the singly charged ion at *m*/*z* 1471.29 corresponding to GD3 (d18:1/18:0) from BMLA. Acquisition time 1 min. Insets: fragmentation schemes of the oligosaccharide core and ceramide moiety. Reprinted with permission from [173].

Unlike HB, the 125 species forming the gangliosidome of BMLA were found to contain mostly short glycan chains with a lower number of Neu5Ac residues attached to the main core, such as GM1, GM2, GM3 and GM4, or even lacking completely the sialylation to yield the GA1 and GA2 type of species. Some of these short-chain gangliosides were found to present modifications by Fuc and *O*-Ac, an unusual feature, which appears as a marker clearly distinguishing the metastatic tissue from the normal one. The BMLA-associated Fuc-GM4, Fuc-GM3, di-*O*-Ac-GM3, *O*-Ac-GM3 fall in this category. On the other hand, previous reports indicated that the structures in this class represent fetal brain markers as well, being developmentally regulated antigens with very low expression in the normal adult brain tissue [63]. Another interesting finding is related to the expression of GD3 species in BMLA, since GD3 was previously reported as involved in the proliferation of tumor cells, influencing the metastatic process and tumor angiogenesis [137,149,174]. For this reason, a part of the research in the field of brain tumor gangliosides is focused on the study of GD3 ganglioside species in relation to the development of immunotherapeutic schemes [61]. However, since detailed knowledge on the structure of the GD3 species implicated in metastasis is necessary as the starting point, chip-based nanoESI MS/MS [173] was applied to the structural elucidation of BMLA-associated GD3. Figure 7 presents MS/MS using the ion at *m*/*z* 1471.29 as the precursor. According to the accurate mass calculation, this ion corresponds to GD3 (d18:1/18:0). Obviously, the optimized sequencing conditions allowed the validation of GD3 structure through the generation of the entire series of glycan-derived fragment ions (B- and Y-type) and a few diagnostic ions, such as S, V, T, P and Q, crucial for the determination of the ceramide configuration (insets Figure 7). By applying CID MS/MS, the structure of the Fuc-GM1 (d18:1/18:0) was also elucidated [173]. Interestingly, the species Fuc-GM1 (d18:1/18:0) associated with BMLA were found to be a rare isomer containing the fucose linked to the internal galactose together with the sialic acid, forming a Neu5Ac-Gal-Fuc motif.

An important technical aspect derived from this study is related to the sensitivity of the employed method. The flow rate delivered by the chip-nanoESI under the working conditions was about 100 nL/min, while the signal was acquired for only 1 min using a sample concentration of 2.5 pmol/μL. Consequently, for the screening experiment, only 250 fmols of sample were used, whereas the complete analysis, including screening and structural elucidation required only 500 fmols. This high sensitivity is crucial for clinical samples since, in such cases, extremely low amounts of material are available for research. Obviously, from the sensitivity point of view, microfluidics-based MS is ideal in studies targeting biomarker discovery in brain metastases.

**Figure 8:**
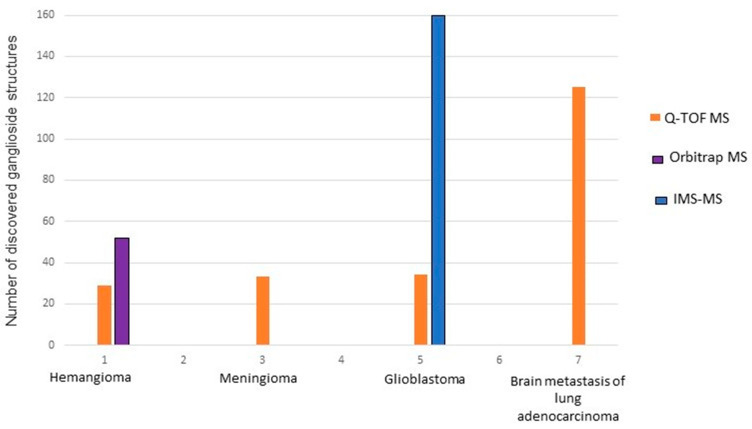
Graphical analysis of the results obtained by different mass spectrometry techniques.

Since in this review we presented various types of MS methods employed in the investigation of gangliosides expressed in a few benign and malignant primary brain tumors as well as secondary brain tumors, Figure 8 depicts a comparative graphical analysis of the performances of these methods in discovery of ganglioside species associated with these tumors.

## 4. Conclusions

As featured in this review, in recent years, several research laboratories have devoted their work to the development of specific and efficient methods for the analysis of gangliosides in brain tumors and expanded the inventory of cancer-relevant structures in the central nervous system. A few ganglioside species were known for some decades to induce the inhibition of cancer cell growth, cell differentiation and/or apoptosis, while a couple of others have been even postulated as specifically associated with primary brain tumors. Since, more recently, ganglioside biosynthesis was found to be severely impacted by the neoplastic transformation and able to upregulate structures with either pro-cancerous or anti-cancerous effects, many of the modern studies are currently focused on the discovery of gangliosides that are valuable indicators for early detection, staging and prognosis of the tumor. On the other hand, some research groups started to be engaged in the exploration of ganglioside structures, which could be used as tumor targets and/or therapeutic agents.

Among all techniques introduced in brain tumor ganglioside analysis and continuously refined for this purpose over the years, mass spectrometry contributed the most to the field. The results highlighted here show that the capability of MS techniques to discover and characterize novel cancer-associated ganglioside species increased constantly over the years. The optimization and introduction in brain tumor ganglioside research of high and ultra-high resolution instruments, such as QTOF, FTICR and Orbitrap MS, as well as the recent employment of microfluidics-MS, IMS MS and MSI systems in combination with efficient ion fragmentation methods, capable of offering comprehensive structural information, paved the way for: (i) the discrimination, identification, and thus correlation with a certain brain tumor of a much larger number of glycoforms; (ii) the enrichment of the previously existing scarce panel of ganglioside biomarkers with a variety of novel glycoforms; (iii) disclosing a highly altered ganglioside expression in brain tumors; (iv) elucidating some of the ganglioside-dependent mechanisms of tumor proliferation; (v) validating the highly abundant species or the uncommon structural isomers, and; (vi) establishing well-defined sets of biomarkers to be further investigated as potential key-molecules in development of new and more efficient therapeutic schemes. A general feature observed in the case of all tumors presented here is an elevated incidence of short-chain glycan species, which exhibit low sialic acid content, together with considerably reduced ganglioside expression and concentration. In the case of malignant transformations, GD3 and its *O*-acetylated variant, GM2 and GM3, as well as Fuc-GM4 and Fuc-GM3 have been postulated as biomarkers of brain metastasis of lung adenocarcinoma, while GD3, GD2, GM3, and *O*-acetylated variants of GD3 were found associated with gliosarcoma and GBM. The investigated ganglioside model of astrocytoma revealed valuable data related to the biomarker role played by hypersialylated species along with GM3 and *O*-Ac and *O*-Fuc glycoforms.

From the assessed data, it appears evident that the MS of tumor gangliosides results in spectra of extreme complexity and a wealth of compositional and structural information. With the advancements in MS technology, increasingly distinct species will continue to be deciphered in the CNS ganglioside extracts, which will require automation in data mining and interpretation. Following the several attempts of constructing computer programs of limited functionality, the next imperative step is the development of software for the interpretation of mass spectra of CNS gangliosides, the validation of software for the accurate determination of gangliosidome of normal human brain and various brain cancers, and the establishment of a user-friendly structural database as a search platform in brain tumor diagnostics.

## Figures and Tables

**Table 1:** Assignment of the ganglioside ions detected by (−) nanoESI Orbitrap MS in cavernous hemangioma. Reprinted with permission from [67].

Nr. crt.	Proposed Structure	*m*/*z* _theor._	*m*/*z* _exp._	Molecular Ion	Mass Accuracy (ppm)	Relative Abundance %
1	GM3 (d18:0/24:0)	645.3958	645.3930	[M-4H^+^+2Na^+^-H_2_O]^2−^	4.3	35.03
2	GM3 (t18:0/24:0)	662.3986	662.3960	[M-4H^+^+2Na^+^]^2−^	3.9	18.51
3	GT1 (d18:1/18:0)	708.3479	708.3472	[M-3H^+^]^3−^	0.9	28.77
4	GD3 (d18:0/16:0)	712.8989	712.9012	[M−2H^+^-H_2_O]^2−^	−3.2	30.83
5	GD3 (d18:1/16:0)	720.8964	720.8964	[M-2H^+^]^2−^	0	34.05
6	GT1 (d18:1/20:0)	717.6917	717.6918	[M-3H^+^]^3−^	−0.1	31.83
7	GT1 (d18:1/22:0)	727.0355	727.0339	[M-3H^+^]^3−^	2.2	29.41
8	GT1 (t18:1/22:1)	731.6952	731.6950	[M-3H^+^]^3−^	0.2	28.77
9	GM2 (d18:1/24:0)	732.9452	732.9510	[M-2H^+^]^2−^	−7.9	33.55
10	GD3 (d18:1/18:1)	733.9121	733.9118	[M-2H^+^]^2−^	0.4	78.73
11	GD3 (d18:1/18:0)	734.9121	734.9122	[M-2H^+^]^2−^	−0.1	59.21
12	*O*-Ac-GT1 (d18:0/22:0)	735.7073	735.7051	[M-3H^+^-H_2_O]^3−^	2.9	52.67
13	GD3 (d18:1/20:0)	748.9277	748.9276	[M-2H^+^]^2−^	0.1	42.85
14	GM1 (d18:1/14:0)	754.8901	754.8914	[M-3H^+^+Na^+^]^2−^	−1.7	35.78
15	GM1 (d18:0/16:0)	758.9226	758.9269	[M-2H^+^]^2−^	−5.6	28.66
16	GD3 (d18:1/22:1)	761.9356	761.9365	[M-2H^+^]^2−^	−1.1	32.73
17	GD3 (d18:1/22:0)	762.9434	762.9432	[M-2H^+^]^2−^	0.2	42.90
18	GM1 (d18:1/18:2)	769.9148	769.9210	[M-2H^+^]^2−^	−8.0	59.21
19	GD3 (d18:1/24:2)	774.9434	774.9432	[M-2H^+^]^2−^	0.2	47.93
20	GD3 (d18:1/24:1)	775.9512	775.9512	[M-2H^+^]^2−^	0.012	59.21
21	GD3 (d18:1/24:0)	776.9590	776.9592	[M-2H^+^]^2−^	−0.2	47.93
22	GM1 (d18:0/18:0)	783.9292	783.9283	[M-3H^+^+Na^+^]^2−^	1.1	35.78
23	GD2 (d18:0/13:0)	793.4598	793.4618	[M-2H^+^-H_2_O]^2−^	−2.5	51.73
24	GM1 (d18:0/18:0)	794.9202	794.9232	[M-4H^+^+2Na^+^]^2−^	−3.7	41.82
25	GM1 (d18:1/20:0)	796.9371	796.9384	[M-3H^+^+Na^+^]^2−^	−1.6	47.12
26	GM1 (d18:0/20:0)	808.9359	808.9434	[M-4H^+^+2Na^+^]^2−^	−9.2	26.78
27	GM1 (d18:0/20:0)	809.9437	809.9441	[M-4H^+^+2Na^+^]^2−^	−0.4	30.60
28	GQ1 (d18:1/20:0)	814.7235	814.7252	[M-3H^+^]^3−^	−2.0	24.76
29	GD3 (d18:1/28:0)	815.9813	815.9850	[M-3H^+^+Na]^2−^	−4.5	29.53
30	GD2 (d18:1/18:0)	827.4464	827.4387	[M-2H^+^-H_2_O]^2^	9.3	30.19
31	GD2 (d18:1/18:0)	836.4517	836.4515	[M-2H^+^]^2−^	0.2	55.21
32	GD2 (d18:1/20:0)	850.4674	850.4671	[M-2H^+^]^2−^	0.3	41.82
33	Fuc-GM1 (d18:1/18:3)	863.918	863.9180	[M-4H^+^+2Na^+^]^2−^	0	87.86
34	GD1 (d18:1/18:0)	917.4781	917.4782	[M-2H^+^]^2−^	−0.1	94.38
35	GD1 (d18:1/20:0)	922.4885	922.4835	[M-2H^+^-H_2_O]^2−^	5.4	36.29
36	GT3 (d18:1/25:1)	928.5067	928.5061	[M-2H^+^]^2−^	0.6	32.64
37	GD1 (d18:1/20:0)	931.4938	931.4938	[M-2H^+^]^2−^	0	100
38	GD1 (d18:1/22:0)	945.5094	945.5081	[M-2H^+^]^2−^	1.3	38.41
39	*O*-Ac-GD1 (d18:1/20:1)	951.4912	951.4899	[M-2H^+^]^2−^	1.3	66.04
40	GD1 (d18:1/24:1)	958.5173	958.5155	[M-2H^+^]^2−^	1.8	17.46
41	Fuc-GT3 (d18:1/20:2)	965.4888	965.4874	[M−2H^+^]^2−^	1.4	12.38
42	Fuc GT3 (d18:1/20:1)	966.4966	966.4971	[M-2H^+^]^2−^	−0.5	24.90
	*O*-Ac-GD1 (d18:1/22:0)	966.5068	966.4971	[M-2H^+^]^2−^	10.0	12.85
43	Fuc-GD1 (d18:0/18:0)	991.5149	991.5089	[M-2H^+^]^2−^	6.0	12.38
44	*O*-Ac-GM4 (d18:1/16:0)	1031.6628	1031.6579	[M-H^+^]^−^	4.7	79.67
45	GT1 (d18:1/14:0)	1034.995	1034.9885	[M-2H^+^]^2−^	6.2	65.37
46	GT1 (d18:1/18:0)	1063.0258	1063.0255	[M-2H^+^]^2−^	0.2	59.92
47	GT1 (d18:1/20:3)	1074.0181	1074.0160	[M−2H^+^]^2−^	1.9	86.69
	GT1 (d18:1/18:0)	1074.0168	1074.0160	[M-3H^+^+Na^+^]^2−^	0.7	86.69
48	GT1 (d18:1/20:0)	1077.0414	1077.0427	[M-2H^+^]^2−^	−1.2	62.77
49	GT1 (d18:1/22:3)	1088.0337	1088.0338	[M-2H^+^]^2−^	−0.09	73.38
	GT1 (d18:1/20:0)	1088.0325	1088.0338	[M-3H^+^+Na^+^]^2−^	−1.1	73.38
50	GT1 (d18:1/22:0)	1091.0571	1091.0479	[M-2H^+^]^2−^	8.4	54.37
51	GT1 (d18:1/24:1)	1115.0559	1115.0584	[M-3H^+^+Na^+^]^2−^	−2.2	68.90
52	GM3 (d18:0/16:0)	1151.7052	1151.7036	[M-H^+^]^−^	1.3	62.77
53	(CH_3_COO^−^) GalNAc GT1 (d18:1/16:2)	1179.049	1179.0457	[M^−^-H^+^]^2−^	2.7	59.92
54	GA1 (d18:1/18:0)	1235.7626	1235.7589	[M-H^+^ -H_2_O]^−^	2.9	49.90
55	GM3 (d18:1/27:0)	1305.8774	1305.8889	[M-H^+^]^−^	−8.8	28.66
56	GM3 (d18:1/27:0)	1327.859	1327.8499	[M-2H^+^+Na^+^]^−^	6.8	78.90
57	GD3 (d18:1/16:0)	1442.801	1442.8043	[M-H^+^]^−^	−2.2	64.76
58	GM1 (d18:1/14:0)	1488.806	1488.8092	[M-H^+^]^−^	−2.1	58.46
59	GM1 (d18:1/22:0)	1600.9312	1600.9337	[M-H^+^]^−^	−1.5	62.07
60	GD2 (d18:1/17:0)	1659.8955	1659.8918	[M-H^+^]^−^	2.2	78.90
61	GalNAc-GM1 (d18:1/8:1)	1745.9323	1745.9223	[M-H^+^]^−^	5.7	58.08
62	GD1 (d18:1/16:0)	1807.9327	1807.9201	[M-H^+^]^−^	6.9	58.45

**Table 2:** Proposed composition of single components in the ganglioside mixture from AcT as detected by (−) nanoESI MS. Reprinted with permission from [132].

*m*/*z* (Monoisotopic) Experimental	*m*/*z* (Monoisotopic) Theoretical	Mass Accuracy (ppm)	Molecular Ion	Proposed Structure
917.47	917.48	10.90	[M-2H]^2−^	GD1, nLD1 or LD1 (d18:1/18:0)
931.33	931.34	10.74	[M-2H]^2−^	GD1, nLD1 or LD1 (d18:1/20:0)
947.33	947.34	10.55	[M-H]^−^	LacCer (d18:0/22:0)
980.19	980.21	20.40	[M+Na-2H]^−^	GM4 (d18:1/14:2)
1019.78	1019.80	19.60	[M-2H]^2−^	GT2 (d18:1/22:2)
1063.31	1063.33	18.81	[M-2H]^2−^	GT1 (d18:1/18:0) or GT1 (d18:0/18:1)
1074.00	1074.02	18.62	[M+Na-3H]^2−^	GT1 (d18:1/18:0)
1098.18	1098.20	18.21	[M-2H]^2−^	GT1 (d18:1/23:0) or GT1 (d18:0/23:1)
1127.42	1127.45	26.61	[M+Na-2H]^−^	GM3 (d18:1/13:2)
1179.71	1179.74	25.42	[M-H]^−^	GM3 (d18:1/18:0)
1224.33	1224.36	24.50	[M-H]^−^	Fuc-GM3 (d18:1/12:0)
1231.01	1231.04	24.37	[M+2Na-4H]^2−^	GQ1 (d18:1/18:0)
1268.66	1268.69	23.64	[M-H]^−^	GM2 (d18:1/10:0)
1382.78	1382.82	28.92	[M-H]^−^	GM2 (d18:1/18:0)
1408.81	1408.85	28.38	[M-H]^−^	GM2 (d18:1/20:1)
1440.74	1440.78	27.75	[M-H]^−^	GD3 (d18:1/16:1)
1470.99	1471.03	27.19	[M-H]^−^	GD3 (d18:1/18:0)
1518.82	1518.86	26.33	[M-H]^−^	GM1 (d18:0/16:0)
1544.78	1544.83	32.36	[M-H]^−^	GM1, nLM1 or LM1 (d18:1/18:0)
1572.80	1572.85	31.78	[M-H]^−^	GM1, nLM1 or LM1 (d18:1/20:0)
1600.86	1600.92	37.47	[M-H]^−^	GM1, nLM1or LM1 (d18:1/22:0)
1700.83	1700.89	35.27	[M+2Na-3H]^−^	GM1 (d18:1/26:0)
1830.33	1830.40	38.25	[M-H]^−^	GT3 (d18:1/23:1)
1835.87	1835.94	38.12	[M-H]^−^	GD1, nLD1or LD1 (d18:1/18:0)
1857.88	1857.95	37.67	[M+Na-2H]^−^	GD1, nLD1 or LD1 (d18:1/18:0)
1885.92	1885.99	37.11	[M+Na-2H]^−^	GD1 (d18:1/20:0)
1916.85	1916.92	36.51	[M-H]^−^	GD1 (d18:1/24:2)
1965.95	1966.02	35.60	[M-H]^−^	GT2 (d18:1/18:1) or GT2 (d18:0/18:2)
1992.46	1992.53	35.12	[M-H]^−^	Hex-HexNAc-nLM1 (d18:1/24:1)
1996.89	1996.96	35.05	[M-H]^−^	GD2–lactone (d18:1/22:2)
2052.52	2052.60	38.96	[M-H]^−^	GT2 (d18:0/24:0)
2098.16	2098.24	38.13	[M-H]^−^	*O*-Ac-GD1 (d18:1/34:2)
2124.12	2124.20	37.66	[M-H]^−^	GT1 (d18:1/18:2) or GT1 (d18:0/18:3)
2385.13	2385.22	37.73	[M+3Na-4H]^−^	GT1 (18:1/32:2)
2524.14	2524.24	39.61	[M+Na-2H]^−^	GQ1 (d18:1/24:0)
2622.46	2622.56	38.12	[M+Na-2H]^−^	GQ1 (d18:1/31:0)
2827.26	2827.37	38.91	[M-H]^−^(-H_2_O)	GP1 (d18:1/28:2)

**Table 3:** Proposed composition of single components in the ganglioside mixture from ST as detected by (−)nanoESI MS. Reprinted with permission from [132].

*m*/*z* (Monoisotopic) Experimental	*m*/*z* (Monoisotopic) Theoretical	Mass Accuracy (ppm)	Molecular Ion	Proposed Structure
872.37	872.38	11.46	[M-2H]^2−^	*O*-Ac-GD2 (d18:1/20:0)
911.30	911.31	10.97	[M-2H]^2−^	GD1, nLD1 or LD1 (d18:1/18:3)
1063.31	1063.33	18.81	[M-2H]^2−^	GT1 (d18:1/18:0) or GT1 (d18:0/18:1)
1175.94	1175.96	17.00	[M-H]^−^	GM3 (d18:1/18:2)
1197.77	1197.80	25.04	[M-H]^−^	GM3 (d18:0/19:0)
1235.78	1235.81	24.29	[M-H]^−^	GM3 (d18:1/22:0)
1306.03	1306.06	22.97	[M-H]^−^	GM3 (d18:1/27:0)
1510.80	1510.84	26.47	[M-H]^−^	*O*-Ac-GD3 (d18:1/18:1)
1644.27	1644.31	24.33	[M-H]^−^	GM1 (d18:0/25:0)
1662.18	1662.22	24.06	[M-H]^−^	GM1 (d18: 1/29: 2)
1684.78	1684.82	23.73	[M-H]^−^	GD2 (d18:1/19:2) or GD2 (d18:0/19:3)
1847.38	1847.42	21.65	[M-H]^−^	GT3 (d18:0/24:0)
1879.05	1879.09 1879.10	21.28 26.60	[M-H]^−^(-H_2_O) [M+Na-2H]^−^	Fuc-GT3 (d18:0/17:0) or *O*-Ac-GT3 (d18:1/22:2)
2024.17	2024.23	29.64	[M+2Na-3H]^−^	GD1 (d18:0/28:0)
2050.55	2050.60	24.37	[M-H]^−^	GT2 (d18:1/24:0) or GT2 (d18:0/24:1)
2072.15	2072.21	28.95	[M-H]^−^	*O*-Ac-GD1 (d18:1/32:1)
2076.13	2076.19	28.90	[M-H]^−^	*O*-Ac-GD1 (d18:0/32:0)
2102.20	2102.26	28.54	[M-H]^−^	*O*-Ac-GD1 (d18:1/34:0)
2112.02	2112.09	33.14	[M-H]^−^	GT1 (d18:1/17:2) or GT1 (d18:0/17:3)
2123.90	2123.97	32.95	[M-H]^−^	GT1 (d18:1/18:2)
2175.73	2175.80	32.16	[M-H]^−^	GT2 (d18:1/33:0)
2186.52	2186.60	36.57	[M-H]^−^	GT1 (d18:0/22:0)
2237.08	2237.16	35.76	[M-H]^−^	GT1 (d18:1/26:1)
2246.90	2246.98	35.60	[M-H]^−^	Fuc-GT1 (d18:0/16:0)
2287.72	2287.80	34.96	[M-H]^−^	Fuc-GT1 (d18:0/20:0)
2346.00	2346.09	38.36	[M-H]-(-H_2_O)	GQ1 (d18:1/13:1)
2385.13	2385.22	37.73	[M+3Na-4H]^−^	GT1 (18:1/32:2)
2472.12	2472.21	36.40	[M-H]^−^	GQ1 (d18:1/22:1)
2582.46	2582.56	38.71	[M-H]^−^(-H_2_O)	GQ1 (d18:1/31:0)
2618.41	2618.51	38.18	[M+2Na-3H]^−^	GQ1 (d18:0/29:0)
2642.52	2642.62	37.83	[M-H]^−^	*O*-Ac-GQ1 (d18:1/31:0)
2673.42	2673.52	37.41	[M-H]^−^	GP2 (d18:1/27:0)
2906.39	2906.50	37.85	[M-H]^−^	Fuc-GP1 (d18:1/23:1)

**Table 4:** Proposed composition of single components in the ganglioside mixture from NT as detected by (−)nanoESI MS. Reprint with permission from [132].

*m*/*z* (Monoisotopic) Experimental	*m*/*z* (Monoisotopic) Theoretical	Mass Accuracy (ppm)	Molecular Ion	Proposed Structure
917.47	917.48	10.90	[M-2H]^2−^	GD1, nLD1 or LD1 (d18:1/18:0)
931.33	931.34	10.74	[M-2H]^2−^	GD1, nLD1 or LD1 (d18:1/20:0)
947.33	947.34	10.55	[M-H]^−^	LacCer (d18:0/22:0)
980.19	980.21	20.40	[M+Na-2H]^−^	GM4 (d18:1/14:2)
1019.78	1019.80	19.60	[M-2H]^2−^	GT2 (d18:1/22:2)
1063.31	1063.33	18.81	[M-2H]^2−^	GT1 (d18:1/18:0) or GT1 (d18:0/18:1)
1074.00	1074.02	18.62	[M+Na-3H]^2−^	GT1(d18:1/18:0)
1098.18	1098.20	18.21	[M-2H]^2−^	GT1 (d18:1/23:0) or GT1 (d18:0/23:1)
1127.42	1127.45	26.61	[M+Na-2H]^−^	GM3 (d18:1/13:2)
1179.71	1179.74	25.42	[M-H]^−^	GM3 (d18:1/18:0)
1224.33	1224.36	24.50	[M-H]^−^	Fuc-GM3 (d18:1/12:0)
1231.01	1231.04	24.37	[M+2Na-4H]^2−^	GQ1 (d18:1/18:0)
1268.66	1268.69	23.64	[M-H]^−^	GM2 (d18:1/10:0)
1382.78	1382.82	28.92	[M-H]^−^	GM2 (d18:1/18:0)
1408.81	1408.85	28.38	[M-H]^−^	GM2 (d18:1/20:1)
1440.74	1440.78	27.75	[M-H]^−^	GD3 (d18:1/16:1)
1470.99	1471.03	27.19	[M-H]^−^	GD3 (d18:1/18:0)
1518.82	1518.86	26.33	[M-H]^−^	GM1 (d18:0/16:0)
1544.78	1544.83	32.36	[M-H]^−^	GM1, nLM1 or LM1 (d18:1/18:0)
1572.80	1572.85	31.78	[M-H]^−^	GM1, nLM1 or LM1 (d18:1/20:0)
1600.86	1600.92	37.47	[M-H]^−^	GM1,nLM1or LM1 (d18:1/22:0)
1700.83	1700.89	35.27	[M+2Na-3H]^−^	GM1 (d18:1/26:0)
1830.33	1830.40	38.25	[M-H]^−^	GT3 (d18:1/23:1)
1835.87	1835.94	38.12	[M-H]^−^	GD1,nLD1or LD1 (d18:1/18:0)
1857.88	1857.95	37.67	[M+Na-2H]^−^	GD1, nLD1 or LD1 (d18:1/18:0)
1885.92	1885.99	37.11	[M+Na-2H]^−^	GD1 (d18:1/20:0)
1916.85	1916.92	36.51	[M-H]^−^	GD1 (d18:1/24:2)
1965.95	1966.02	35.60	[M-H]^−^	GT2 (d18:1/18:1) or GT2 (d18:0/18:2)
1992.46	1992.53	35.12	[M-H]^−^	Hex-HexNAc-nLM1 (d18:1/24:1)
1996.89	1996.96	35.05	[M-H]^−^	GD2-lactone (d18:1/22:2)
2052.52	2052.60	38.96	[M-H]^−^	GT2 (d18:0/24:0)
2098.16	2098.24	38.13	[M-H]^−^	*O*-Ac-GD1 (d18:1/34:2)
2124.12	2124.20	37.66	[M-H]^−^	GT1 (d18:1/18:2) or GT1 (d18:0/18:3)
2385.13	2385.22	37.73	[M+3Na-4H]^−^	GT1 (18:1/32:2)
2524.14	2524.24	39.61	[M+Na-2H]^−^	GQ1 (d18:1/24:0)
2622.46	2622.56	38.12	[M+Na-2H]^−^	GQ1 (d18:1/31:0)
2827.26	2827.37	38.91	[M-H]^−^(-H_2_O)	GP1 (d18:1/28:2)

**Table 5:** Ganglioside and asialo-ganglioside species in the gangliosidome of BMLA identified by (-) nanoESIchip QTOF MS. Reprinted with permission from [173].

*m*/*z* (Monoisotopic) Theoretical	*m*/*z* (Monoisotopic) Experimental	Mass Accuracy (ppm)	Molecular Ion	Proposed Structure
875.19	874.91	33	[M-H]^−^	LacCer (d18:1/17:0)
933.31	932.99	35	[M-H]^−^	LacCer (d18:0/21:0)
947.34	947.19	16	[M-H]^−^	LacCer (d18:0/22:0)
949.22	949.24	21	[M+2Na-3H]^−^	LacCer (d18:0/19:0)
964.24	963.90	35	[M-H]^−^	GM4 (d18:0/14:0)
982.19	981.94	25	[M+Na-2H]^−^	GM4 (d18:1/14:1)
984.21	983.87	34	[M+Na-2H]^−^	GM4 (d18:1/14:0) or GM4 (d18:0/14:1)
1122.48	1122.23	22	[M-H]^−^	GA2 (d18:0/20:0)
1138.44 1138.48	1138.15	25 29	[M-H]^−^	Fuc-GM4 (d18:0/16:0) GA2 (t18:0/20:0)
1150.49 1150.40	1150.17	28 20	[M-H]^−^ [M-H]^−^	GA2 (d18:1/21:0) GM3 (d18:1/16:1)
1168.42	1168.01	35	[M-H]^−^	GM3 (t18:0/16:0)
1178.46	1178.14	27	[M-H]^−^	GM3 (d18:1/18:1)
1179.74	1180.10	30	[M-H]^−^	GM3 (d18:1/18:0)
1182.49	1182.21	24	[M-H]^−^	GM3 (d18:0/18:0)
1184.37	1184.08	24	[M-H]^−^	*O*-Ac-GA1 (d18:1/10:0)
1194.50	1194.15	29	[M-H]^−^	GM3 (d18:1/19:0) or GM3 (d18:0/19:1)
1206.51 1206.64	1206.33	15 26	[M-H]^−^ [M-H]^−^	GM3 (d18:1/20:1) GA2 (d18:0/26:0)
1222.51 1222.55	1222.19	26 29	[M-H]^−^ [M-H]^−^	*O*-Ac-GM3 (d18:1/18:0) GM3 (d18:0/21:1) or GM3 (d18:1/21:0)
1234.56 1234.52	1234.22	27 24	[M-H]^−^	GM3 (d18:1/22:1) *O*-Ac-GM3 (d18:1/19:1)
1248.55 1248.59	1248.18	33 30	[M-H]^−^	*O*-Ac-GM3 (d18:1/20:1) GM3 (d18:1/23:1)
1248.59	1249.02	34	[M-H]^−^	GM3 (d18:1/23:0)
1260.60	1260.33	21	[M-H]^−^	GM3 (d18:1/24:2)
1262.62	1262.35	21	[M-H]^−^	GM3 (d18:1/24:1)
1264.63	1264.19	35	[M-H]^−^	GM3 (d18:1/24:0)
1276.61 1276.64 1276.69	1277.01	31 29 25	[M-H]^−^ [M-H]^−^ [M-H]^−^(-H_2_O)	*O*-Ac-GM3 (d18:1/22:0) GM3 (d20:1/23:1) GM3 (d18:0/26:0)
1278.66 1278.61	1278.21	35 31	[M-H]^−^ [M-H]^−^	GM3 (d20:1/23:0) *O*-Ac-GM3 (d18:1/22:0) or *O*-Ac-GM3 (d18:0/22:1)
1288.67 1288.67	1289. 04	29 29	[M-H]^−^ [M-H]^−^	GM3 (d18:1/26:2) or GM3 (d18:2/26:1) GM3 (d20:1/24:2)
1292.68	1292.23	35	[M-H]^−^	GM3 (d18:1/26:0) or GM3 (d18:0/26:1)
1296.54 1296.59 1296.61	1296.24	23 27 28	[M-H]^−^ [M-H]^−^ [M-H]^−^	Fuc-GM3 (d18:1/16:1) *O*-Ac-GA1 (d18:1/18:0) GA1 (d18:0/21:0) or GA1 (d18:0/21:0)
1405.65	1405.21	31	[M+Na-2H]^−^	GM2 (d18:1/18:0)
1420.68	1420.80	8	[M-H]^−^	*O*-Ac-GM2 (d18:2/18:2)
1435.59	1435.21	26	[M+Na-2H]^−^	GD3 (d18:1/14:1) or GD3 (d18:0/14:2) or GD3 (d18:2/14:0)
1441.66	1441.19	33	[M-H]^−^	GD3 (d18:1/16:1) or GD3 (d18:0/16:2) or GD3 (d18:2/16:0)
1471.73	1471.28	31	[M-H]^−^	GD3 (d18:1/18:0)
1493.71	1493.23	32	[M+Na-2H]^−^	GD3 (d18:1/18:0)
1515.69 1515.74 1515.78	1515.29	26 30 32	[M+2Na-3H]^−^ [M-H]^−^ [M-H]^−^	GD3 (d18:1/18:0) or GD3 (d18:0/18:1) GM1 (d18:1/16:1) or GM1 (d18:0/16:2) or GM1 (d18:2/16:0) *O*-Ac-GD3 (d18:0/18:0)
1515.71 1515.75	1516.01	20 17	[M + Na-2H]^−^ [M-H]^−^	GD3 (d18:1/20:2) or GD3 (d18:0/20:3) or GD3 (d18:2/20:1) GM1 (d18:2/16:0) or GM1 (d18:1/16:1) or GM1 (d18:0/16:2)
1527.83	1528. 16	22	[M-H]^−^	GD3 (d18:0/22:0)
1541.79	1542. 19	26	[M-H]^−^	GM1 (d18:1/18:2) or GM1 (d18:2/18:1) or GM1 (d18:0/18:3)
1569.78 1569.83 1569.77	1570.29	32 29 33	[M+2Na-3H]^−^ [M-H]^−^ [M+Na-2H]^−^	GD3 (d18:1/22:0) or GD3 (d18:0/22:1) GM1 (d18:1/20:1) or GM1 (d18:0/20:2) or GM1 (d18:2/20:0) GD3 (d18:0/24:2) or GD3 (d18:1/24:1) or GD3 (d18:2/24:0)
1597.88	1598.09	13	[M-H]^−^	GM1 (d18:0/22:2) or GM1 (d18:1/22:1) or GM1 (d18:2/22:0)
1611.77	1612.17	25	[M+2Na-3H]^−^	GM1 (d18:1/20:2)
1625.89 1625.92	1625.40	30 30	[M+2Na-3H]^−^ [M-H]^−^	GD3 (d18:1/26:1) or GD3 (d18:0/26:2) or GD3 (d18:2/26:0) GM1 (d18:1/24:2)
1627.90 1626.93	1627.41	30 29	[M+2Na-3H]^−^ [M-H]^−^	GD3 (d18:0/26:1) or GD3 (d18:1/26:0) GM1 (d18:0/24:2) or GM1 (d18:1/24:1) or GM1 (d18:2/24:0)
1629.92 1628.94	1629.42	31 29	[M-H]^−^ [M-H]^−^	GM1 (d18:0/24:1) or GM1 (d18:1/24:0) di-*O*-Ac-GM1 (d18:1/18:0)
1659.79	1660.18	23	[M+3Na-4H]^−^	GM1 (d18:1/22:3) or GM1 (d18:0/22:4) or GM1 (d18:2/22:2)
1674.87	1675.23	21	[M+Na-2H]^−^ (-H_2_O)	GD2 (d18:1/18:2)
1748.97	1749.39	24	[M+Na-2H]^−^	GD2 (d18:1/22:1)
1766.97	1767.28	18	[M-H]^−^ (-H_2_O)	GT3 (d18:1/20:1)
1785.07	1785.37	17	[M-H]^−^	*O*-Ac-GD2 (d18:1/23:0) or *O*-Ac-GD2 (d18:0/23:1)
1833.81 1833.07	1833.28	29 11	[M-H]^−^ [M-H]^−^	GT3 (d18:0/23:0) *O*-Ac-GT3 (d18:0/20:0)
1861.12 1861.12	1861.24	6 6	[M-H]^−^ [M-H] (-H_2_O)	*O*-Ac-GT3-lactone (d18:0/22:0) *O*-Ac-GT3 (d18:0/22:0)
1879.09 1879.10 1879.99	1879.39	16 15 32	[M+Na-2H]^−^ [M-H]^−^(-H_2_O) [M-H]^−^	*O*-Ac-GT3 (d18:2/22:1) Fuc-GT3 (d18:0/17:0) GT2 (d18:1/12:1) or GT2 (d18:2/12:0)
1909.16	1909.03	7	[M-H]^−^	GD1 (d18:1/22:0)
1960.21 1960.12	1959.84	19 14	[M-H]^−^ (-2H_2_O) [M-H]^−^	GT2 (d18:0/20:0) GT2 (d18:1/18:3) or GT2 (d18:2/18:2)
1990.17 1990.19	1989.78	20 21	[M+Na-2H]^−^ [M-H]^−^	GT2 (d18:0/18:0) GT2 (d18:0/20:3) or GT2 (d18:1/20:2) or GT2 (d18:2/20:1)
1990.19	1990.83	32	[M-H]^−^	GT2 (d18:1/20:1) or GT2 (d18:0/20:2) or GT2 (d18:2/20:0)
2005.20 2006.19	2005.63	21 28	[M-H]^−^	Fuc-GD1 (d18:1/20:2) *O*-Ac-GT2 (d18:1/18:1)
2048.23 2048.10	2048.80	28 34	[M-H]^−^ [M-H]^−^(-H_2_O)	di-*O*-Ac-GT2 (d18:0/18:0) GT1 (d18:2/14:2) or GT1 (d18:3/14:1)
